# Quantifying dissipation in actomyosin networks

**DOI:** 10.1098/rsfs.2018.0078

**Published:** 2019-04-19

**Authors:** Carlos Floyd, Garegin A. Papoian, Christopher Jarzynski

**Affiliations:** 1 Biophysics Program, University of Maryland, College Park, MD 20742, USA; 2 Department of Chemistry and Biochemistry, University of Maryland, College Park, MD 20742, USA; 3 Institute for Physical Science and Technology, University of Maryland, College Park, MD 20742, USA; 4 Department of Physics, University of Maryland, College Park, MD 20742, USA

**Keywords:** dissipation, actomyosin networks, network percolation, mean-field modelling, active matter, entropy production

## Abstract

Quantifying entropy production in various active matter phases will open new avenues for probing self-organization principles in these far-from-equilibrium systems. It has been hypothesized that the dissipation of free energy by active matter systems may be optimized, leading to system trajectories with histories of large dissipation and an accompanying emergence of ordered dynamical states. This interesting idea has not been widely tested. In particular, it is not clear whether emergent states of actomyosin networks, which represent a salient example of biological active matter, self-organize following the principle of dissipation optimization. In order to start addressing this question using detailed computational modelling, we rely on the MEDYAN simulation platform, which allows simulating active matter networks from fundamental molecular principles. We have extended the capabilities of MEDYAN to allow quantification of the rates of dissipation resulting from chemical reactions and relaxation of mechanical stresses during simulation trajectories. This is done by computing precise changes in Gibbs free energy accompanying chemical reactions using a novel formula and through detailed calculations of instantaneous values of the system’s mechanical energy. We validate our approach with a mean-field model that estimates the rates of dissipation from filament treadmilling. Applying this methodology to the self-organization of small disordered actomyosin networks, we find that compact and highly cross-linked networks tend to allow more efficient transduction of chemical free energy into mechanical energy. In these simple systems, we observe that spontaneous network reorganizations tend to result in a decrease in the total dissipation rate to a low steady-state value. Future studies might carefully test whether the dissipation-driven adaptation hypothesis applies in this instance, as well as in more complex cytoskeletal geometries.

## 1. Introduction

The actin-based cytoskeleton is a dynamic supramolecular structure that, by sustaining and releasing mechanical stress in response to various physiological cues, mediates the exertion of force by cells both on their environments and within their bodies [1,2]. These cytoskeletal structures are composed of long (of the order of 1 μm *in vivo* [3]) actin polymers which are interconnected by various cross-linkers, as well as by myosin motor filaments, resulting in a three-dimensional network-like organization referred to as an ‘actomyosin network’ [4,5].^1^ Part of the intricacy of actomyosin network dynamics is due to the mechanosensitive kinetic reaction rates controlling cross-linker and myosin filament unbinding as well as myosin filament walking: at high tension, cross-linkers will unbind more quickly (slip-bond), whereas motors will unbind and walk less quickly (catch-bond and stalling) [6–8]. These reactions control the actomyosin network connectivity, which, in turn, determines the ability of the network to globally distribute stress [9]. Thus the mechanosensitive feedback introduces nonlinear coupling between the stress sustained by an actomyosin network and the network’s ability to reorganize in response to that stress. In order to be responsive to physiological cues, the dynamics of these systems occur far from thermodynamic equilibrium; the hydrolysis of an out-of-equilibrium concentration of ATP molecules fuels (a) the stress-generating activity of the myosin motor filaments and (b) filament treadmilling [10–14]. Filament treadmilling is a steady-state situation in which the polymerization at the plus end of the filament is compensated by the depolymerization at the minus end, resulting in the filament moving forward without its length changing. As a result of these local free energy-consuming processes, actomyosin networks constitute an interesting and biologically important example of soft active matter. Active matter is composed of agents that individually transduce free energy from some external source, in this case, the chemical potential energy of many ATP molecules [15–17]. Dissipation in these systems results when the free energy consumed Δ*G* is greater than the quantity of work *W* done by the system on its environment, with the remainder Δ*G* − *W* serving to increase the total entropy.

The viewpoint of actomyosin networks as active matter systems has been fruitfully adopted in recent theoretical and experimental studies, yet a lack of ability to quantify the rates of free energy transduction by these systems has hindered development of some of these lines of study. The emergence of distinctive dynamical states (for instance pulsing actin waves or vortices) during the self-organization of actomyosin systems has been documented in several *in vitro* experiments [18–20]. These emergent patterns depend sensitively on the concentrations of myosin filaments and cross-linkers: myosin filament concentration controls the level of active stress generation, and cross-linker concentration controls the degree of mechanical coupling of actin filaments, which has been described using the language of percolation theory [9,21]. While these emergent dynamic patterns have been characterized in detail, a general mechanism explaining why these patterns emerge under given conditions has not yet been proved. It might be expected, given that these systems operate away from thermodynamic equilibrium, that the quantity of free energy dissipated during a system’s evolution is optimized, similar to the principle of minimum entropy production in the near-equilibrium theory of irreversible thermodynamics [22]. However, this minimum entropy production principle breaks down in the far-from-equilibrium, nonlinear-response regime, where many active matter systems including actomyosin networks operate [23]. It has recently been proposed that another optimization principle applies arbitrarily far from equilibrium. This idea, referred to as dissipation-driven adaptation, suggests that, with other relevant factors being held equal, a coarse-grained trajectory of some non-equilibrium system will be more likely than other alternatives if the amount of free energy dissipated along that trajectory is maximal [24–26]. This organizing principle has been borne out in model systems [27,28], yet has also been shown to have certain counter examples [29]; it remains actively debated. In the case of actomyosin systems, it has not yet been tested because of the difficulty in measuring dissipated free energy using most experimental approaches. In this paper, we take the first steps towards such a test, by developing a simulation methodology allowing the quantification of dissipated free energy during the self-organization of actomyosin networks.

In addition to being of interest in the field of active matter systems, dissipation in actomyosin networks has also been an important factor in recent experimental studies of cell mechanics. Rheological properties of actomyosin networks largely determine rheological properties of the whole cell, and it has been discovered that the dissipative component of the cell’s viscoelastic response to mechanical oscillations (called the loss modulus) is partly attributable to the ATPase activity of myosin motor filaments in actomyosin cytoskeleton [30–32]. In the context of cell mechanics, myosin filaments have several roles: they produce mechanical stress by pulling on actin filaments, they dissipate mechanical stress by disassembling actin filaments and higher order stress-sustaining filament structures, and they consume chemical energy through the hydrolysis of ATP molecules. It has been proposed that a lack of detailed understanding of the effects of these processes, and of dissipation in actomyosin systems more generally, underlies inconsistent, widely variable traction force microscopy measurements of cell migration [33]. Progress along this line is hindered by the absence of methods to study dissipation in actomyosin networks directly and at sufficiently high spatio-temporal resolution.

To address these needs, we introduce a computational approach to measure dissipation during simulations of actomyosin network self-organization using the simulation platform MEDYAN (mechanochemical dynamics of active networks) [34,35]. MEDYAN simulations marry stochastic reaction–diffusion chemistry algorithms with detailed mechanical models of actin filaments, cross-linkers, myosin motor filaments and other associated proteins, and it also accounts for mechanosensitive reaction rates. This combination of simulation features makes this software uniquely capable of probing the complexity of actomyosin network dynamics. For instance, past studies using MEDYAN have investigated the dependence of network collapse on myosin filament and cross-linker concentrations, as well as the origin of local contractility in actomyosin networks [34,36]. We refer the reader to the paper describing MEDYAN for a detailed discussion of the various aspects of the simulation platform [34], while here we describe an extension of that platform that allows for calculation of the energetics of the chemical and mechanical events occurring during simulation. We use these new capabilities to characterize the dissipation resulting from filament treadmilling, for which we further introduce a mean-field model, as well as from myosin filament walking. We study both the time-dependence and the distributions of dissipation rates as concentrations of cross-linkers and myosin filaments are varied, observing that transduction of chemical energy to stored mechanical energy is more efficient at denser network organizations. For these simulations, we first explore systems with ‘plain’ myosin filaments and cross-linkers that are not mechanosensitive, in order to simplify the overall dynamics. We then introduce their mechanochemical coupling to understand its effect on the observed trends. We end by discussing how this new methodology can provide a valuable technique to advance the studies of actomyosin networks mentioned above.

## 2. Methods

### 2.1. Measuring dissipation in MEDYAN

We first give a brief overview of the MEDYAN simulation platform. MEDYAN employs a stochastic chemical evolution algorithm, in conjunction with mechanical representations of polymers and cross-linking proteins, to simulate the dynamics of networks with active components, including but not limited to actomyosin networks. The simulation space comprises a grid of reaction–diffusion compartments, inside which chemical species (e.g. unpolymerized subunits or cross-linking proteins) are assumed to be homogeneously distributed without specified locations, and which participate in reactions (e.g. (de)polymerization or (un)binding) according to mass-action kinetics; the species can additionally jump between compartments in diffusion events. When an unpolymerized subunit polymerizes to or nucleates a filament, it becomes part of the mechanical subsystem, gaining location coordinates in the simulation volume and becoming subject to mechanical potentials depending on its interaction with other mechanical elements. Through chemical reactions such as myosin filament binding and walking, the mechanical energy of the system changes, and the new net forces are then periodically relaxed in a mechanical equilibration phase, using conjugate gradient minimization. We fill in salient details of the above overview as they become relevant below. A user provides input data including system size and simulation length, mechanical parameters (e.g. stretching and bending constants and excluded volume cutoff distances), size of the polymer subunits, energy minimization algorithm parameters, chemical simulation algorithm parameters, choices for the modelling of force-sensitive reaction rates, initial conditions of the filaments (either specified or randomly generated), a list of reacting species and their associated parameters, a list of reactions involving those species and their associated parameters, and a list of desired output information. The output of a simulation is a set of trajectory files containing information at each time point, which can include positions of the mechanical network elements, tensions on the elements, and copy numbers of the chemical species, among other things. In the electronic supplementary material, we discuss parametrization of the simulations analysed in this paper. MEDYAN is extensible in that it is possible to implement new types of outputs, depending on the experimental needs; in this paper, we describe a novel output that reports the changes in the Gibbs free energy of the system.

As a MEDYAN simulation progresses, the Gibbs free energy of the system continually changes due to occurrences of chemical reactions and structural rearrangements of the polymer network. These processes are driven by an out-of-equilibrium concentration of ATP which fuels filament treadmilling and myosin filament walking. Dissipation measurement in MEDYAN works by calculating running totals of the chemical and mechanical energy changes. The running totals can then be converted into instantaneous rates by taking the numerical derivative at each time point using the forward difference quotient. The algorithm for tracking these energy changes is compatible with the following sequence of consecutive procedures that make up one iterative cycle of a MEDYAN simulation [34]:

(1) Evolve system with stochastic chemical simulation for time *t*_min_.
(2) Calculate the changes in the mechanical energy resulting from the reactions in Step 1.
(3) Mechanically equilibrate the network based on the new stresses calculated in Step 2.
(4) Update the reaction rates of force-sensitive reactions based on the new forces.

**Figure 1.**
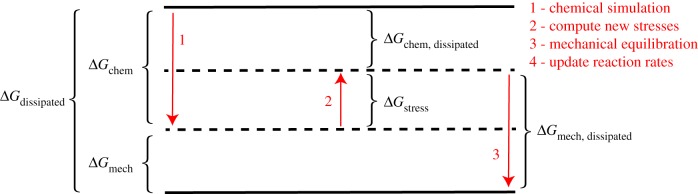
Energy-level diagram indicating which changes in free energy are tabulated during the four procedures constituting one cycle in MEDYAN simulation. Step 4, mechanochemical update of reaction rates, does not result in a free energy change. Dotted lines represent energy levels that are intermediate during the iterative cycle, and solid lines represent the energies at the beginning and end of one cycle. (Online version in colour.)

Dissipation tracking is done by calculating for each of these four steps a change in free energy, and then adding these free energy changes to determine the total change in free energy resulting from each cycle, Δ*G*_dissipated_. Since, at least in this study, the actomyosin network is not mechanically coupled to any work reservoir external to the simulation volume (i.e. *W* = 0), Δ*G*_dissipated_ is indeed dissipated energy [37]. This methodology could be straightforwardly extended to account for work exchanged with an external system in future studies, however. Step 4 in the MEDYAN simulation cycle does not result in a change in free energy, as explained in the electronic supplementary material. The notation chosen for these free energy changes, as well as the direction in which the energy is changing during each procedure, is illustrated in figure 1.

From this picture, we have the following relations:

2.1 $$\Delta G_{\mathrm{dissipated}}=\Delta G_{\mathrm{chem}}+\Delta G_{\mathrm{mech}},$$

2.2 $$\Delta G_{\mathrm{dissipated}}=\Delta G_{\mathrm{chem},\;\mathrm{dissipated}}+\Delta G_{\mathrm{mech},\;\mathrm{dissipated}},$$

2.3 $$\Delta G_{\mathrm{chem},\;\mathrm{dissipated}}=\Delta G_{\mathrm{chem}}+\Delta G_{\mathrm{stress}}$$

2.4 $$\text{and}\;\;\;\Delta G_{\mathrm{mech},\;\mathrm{dissipated}}=\Delta G_{\mathrm{mech}}-\Delta G_{\mathrm{stress}}.$$

For the depicted relative position of energy levels, the sign convention is such that all values of Δ*G* except for Δ*G*_stress_ will be negative (indicated by the arrow’s direction), since *G* refers to the free energy of the system, not of its environment, and will therefore tend to be negative as the system moves down the free energy landscape. The usual intuition that the total dissipation is positive can be stated

2.5 $$\Delta G_{\mathrm{dissipated}}^{+}=-\Delta G_{\mathrm{dissipated}}>0,$$

where the superscript ‘+’ indicates the positive change in the total entropy.

Equation (2.3) says that, given some change in the system’s chemical potential energy, Δ*G*_chem_, resulting from reactions occurring during Step 1, a portion of that energy is used to deform the polymer network (e.g. via myosin filament pulling on actin filaments). This increases the mechanical energy in the network by an amount Δ*G*_stress_. Only the portion of Δ*G*_chem_ which has not gone into Δ*G*_stress_ has been dissipated as heat. In Step 3, the network is mechanically equilibrated, resulting in relaxation of net forces (though not of all stresses) and updating of the network elements’ positions. We refer to the decrease in mechanical energy resulting from this relaxation as Δ*G*_mech, dissipated_.

The calculation of Δ*G*_stress_ and Δ*G*_mech, dissipated_ is based on a set of mechanical potentials describing interactions between elements of the actomyosin network. Polymers are modelled as a sequence of thin, unbendable, yet extensible cylinders that are joined at their ends by beads whose positions define the polymer’s configuration. The structural resolution of MEDYAN is at the level of the cylinders, which in this study are 27 nm long and have effective diameters of approximately 5 nm; however, the diameter is not a parameter of the simulation, being instead effectively determined by the strength of the excluded volume interaction between cylinders. Cross-linking proteins (e.g. *α*-actinin and myosin filaments) are modelled as Hookean springs connecting these cylinders by attaching to discrete binding sites. Included among the mechanical potentials are various modes of filament deformation, excluded volume interactions, and stretching of cross-linkers and myosin filaments. Mechanical equilibration is accomplished by constrained minimization of the mechanical energy with respect to the positions of the network elements. A full description of the mechanical potentials and equilibration protocols is given in [34]. Determining Δ*G*_stress_ and Δ*G*_mech, dissipated_ requires evaluating the instantaneous total mechanical energy of the system at certain points during the iterative simulation cycle and taking the difference of those values.

The calculation of Δ*G*_chem_ and Δ*G*_chem, dissipated_ is accomplished by incrementing a running total of the chemical energy *G*_chem_ whenever a reaction stochastically occurs during Step 1, and finding the accumulated change at the end of the protocol. Chemical stochastic simulation in MEDYAN uses a Gillespie-like reaction–diffusion algorithm over a grid of compartments which constitutes the simulation volume. Diffusing species are assumed to be homogeneous (i.e. obey mass-action kinetics) inside the compartments, and can jump between the compartments leading to concentration gradients at the scale of the compartment length (taken to be roughly the Kuramoto length of diffusing G-actin, following [35]). The evolving polymer network is overlaid on this compartment grid, with each piece of a polymer reacting with diffusing species according to the concentrations in its local compartment. Again, we refer the reader to [34] for a more detailed description of the chemical dynamics. For the present purpose of measuring dissipation, we introduced into this simulation protocol a precise formula for the change in Gibbs free energy corresponding to the occurrence of various reactions as a function of the instantaneous compartment concentrations. In the electronic supplementary material, we establish this formula, while we present its derivation in an accompanying paper [38]. The set of chemical reactions used to describe actomyosin networks in this study is based on a previous model of actin polymerization dynamics that explicitly treats hydrolysis states of the nucleotide bound to each actin subunit [39,40]. This level of detail allows us to quantify the dissipation resulting from ATP hydrolysis during filament treadmilling. To increase computational efficiency, we neglect the dynamics of nucleotide hydrolysis states of the tips of the filaments. This has been shown in previous work to be a valid approximation to the full dynamics which includes the states of the filament tips [40]. We refer to the resulting set of reactions describing actin polymerization dynamics as the constant tip (CT) model. However, unlike in the original CT model, here we explicitly include G-actin bound to ADP-Pi as a reacting species, for completeness and since the extra computational strain of doing so is small. The actin subunit species tracked in this model are distinguished by their polymerization state and by the hydrolysis states of the nucleotide to which they are bound. We notate a species as *G* or *F*, to represent globular (un-polymerized) or filamentous (polymerized) actin, respectively, superscripted by *T*, *Pi* or *D* to represent that it is bound to ATP, ADP-Pi or ADP, respectively; thus for instance filamentous actin bound to ADP-Pi is notated *F*^*Pi*^. We also include reactions describing cross-linker (un)binding and myosin filament (un)binding and walking. We exclude filament nucleation, severing, destruction and annealing reactions, thus the number of filaments is constant throughout the simulation trajectories. In the electronic supplementary material, we describe how we compute the change in Gibbs free energy for each reaction in this set, as well as how we parametrize the simulations.

Finally, we developed a mean-field model to describe just the dissipation resulting from reactions in the CT model, i.e. excluding cross-linkers and myosin filaments. We describe the model and present its results in the electronic supplementary material. We find, among other things, that the steady-state dissipation rate from filament treadmilling counter-intuitively does not depend on the total amount of actin, but only on the number of filaments.

## 3. Results

### 3.1. Total dissipation rates of disordered networks do not increase

**Figure 2.**
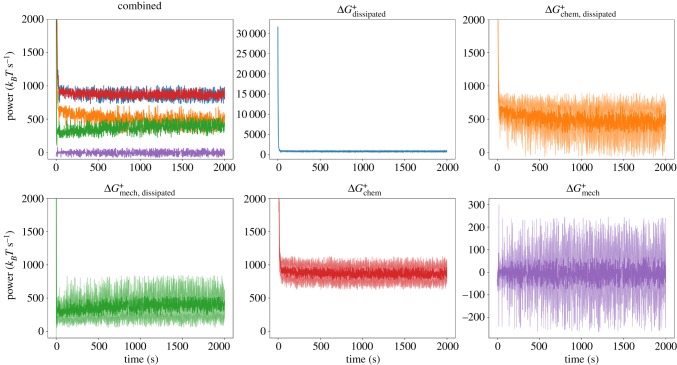
Top left: Combined trajectories of five quantities tracked during a MEDYAN simulation, averaged over 10 separate runs. The colour coding is indicated by the remaining panels. Note the close overlap between $\Delta G_{\mathrm{dissipated}}^{+}$ and $\Delta G_{\mathrm{chem}}^{+}$. In the remaining panels, the individual trajectories are visualized with their standard deviations at each time point over the 10 runs visualized as lighter curves above and below the main curve. In the plots for $\Delta G_{\mathrm{dissipated}}^{+}$ and $\Delta G_{\mathrm{mech}}^{+}$, the full range is visualized; however, this range is cropped in the other plots to aid visibility. (Online version in colour.)

We studied dissipation rates accompanying the process of myosin-driven network self-organization. We first excluded in these simulations the force-sensitivity of the reaction rates describing cross-linker unbinding and myosin filament unbinding and walking. This allowed us to understand a simplified version of the dynamics (we later discuss the effect of including mechanochemical feedback). We analysed the trajectories of the quantities Δ*G*_dissipated_, Δ*G*_chem, dissipated_, Δ*G*_mech, dissipated_, Δ*G*_chem_, and Δ*G*_mech_ over a set of simulations with identical initial concentrations but different random filament distributions. In figure 2, we display these trajectories averaged over 10 runs. We used a simulation volume of 1 μm^3^, divided into eight compartments, and initial conditions of equal amounts (10 μM each) of *G*^*T*^ and *G*^*D*^ actin in a 0.08 μM pool of seed filaments containing *F*^*T*^, as well as 0.2 μM myosin and 1.0 μM cross-linkers. Similar trajectories for other conditions are shown in electronic supplementary material, figures S6 and S7. Points lying outside three median absolute deviations (MADs) have been excluded for this visualization [41].^2^

**Figure 3.**
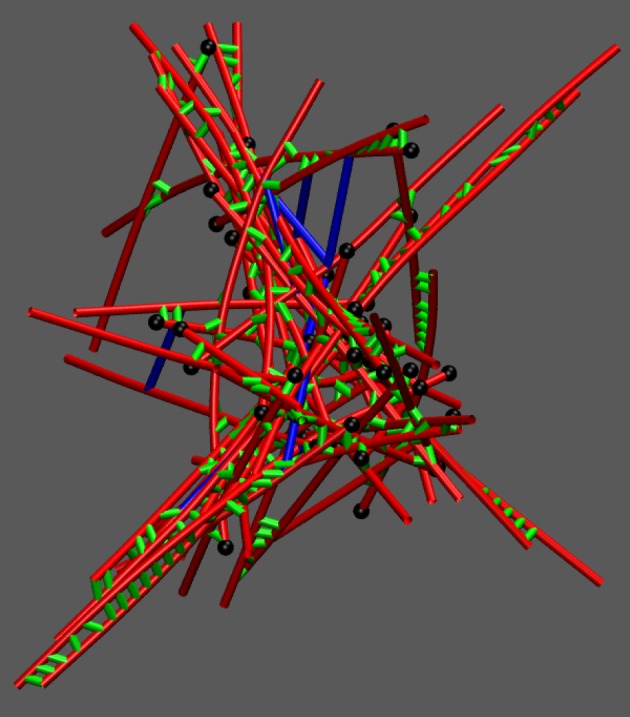
Snapshot of a percolated actomyosin network in MEDYAN under conditions *C*_CL_ = 5.0 μM and *C*_M_ = 0.4 μM. Actin filaments are drawn in red, cross-linkers are drawn in green, myosin filaments are drawn in blue, and the plus ends of filaments are drawn as black spheres. (Online version in colour.)

**Figure 4.**
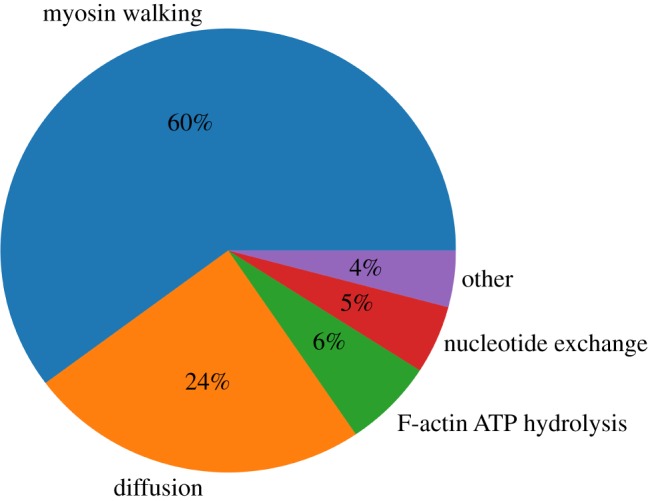
Integrated contributions of each reaction in MEDYAN to the total Δ*G*_chem_ along a simulation trajectory with *C*_CL_ = 1.0 μM and *C*_M_ = 0.2 μM. (Online version in colour.)

For each tracked quantity, there is an initial transient phase (lasting a few tens of seconds) followed by fluctuations around a roughly steady value; we do not observe in this set of simulations any slow approach to a significantly different total dissipation rate, which might correspond to large reorganizations of the network. However, for high concentrations of cross-linkers (*C*_CL_ = 5.0 μM) and myosin (*C*_M_ = 0.4 μM), we observe that the contribution of $\Delta G_{\mathrm{mech},\;\mathrm{dissipated}}^{+}$ to $\Delta G_{\mathrm{dissipated}}^{+}$ tends to increase relative to that of $\Delta G_{\mathrm{chem},\;\mathrm{dissipated}}^{+}$ (electronic supplementary material, figure S7). It is known that network percolation and collapse occurs only under certain conditions of myosin and cross-linker concentrations [21,34], and it is under these conditions which result in collapse that we observe an increase in $\Delta G_{\mathrm{mech},\;\mathrm{dissipated}}^{+}$ relative to $\Delta G_{\mathrm{chem},\;\mathrm{dissipated}}^{+}$ (electronic supplementary material, figures S8–S10). This indicates that more mechanical stress is being created by myosin filament walking as the network collapses and becomes more densely cross-linked. The transient phase corresponds to the initial polymerization of the seed filaments followed by the initial mechanical coupling of filaments by cross-linkers and myosin filaments. Following the transient phase, the networks in this study are generally disordered (figure 3). The dissipation rate corresponding to the initial polymerization of the seed filaments is much larger than the chemical dissipation resulting from myosin filament activity. However, following the transient phase, after which the treadmilling dissipation has reached its steady state (electronic supplementary material, figure S2), the contribution from myosin filament activity outweighs the dissipation resulting from filament treadmilling. Tracking the instantaneous rate of change in $\Delta G_{\mathrm{chem}}^{+}$ resulting from each reaction separately, we observe myosin filament walking to contribute the majority to $\Delta G_{\mathrm{chem}}^{+}$ after the transient phase; however, the amount depends on *C*_CL_ and *C*_M_. The integrated contributions of each reaction to $\Delta G_{\mathrm{chem}}^{+}$ for different conditions are shown in figure 4 and electronic supplementary material, figures S11 and S12. Interestingly, diffusion contributes an appreciable fraction to $\Delta G_{\mathrm{chem}}^{+}$; plus and minus ends of actin filaments tend to localize together (figure 3) and through treadmilling deplete the concentrations of *G*^*T*^ and *G*^*D*^ in certain reaction compartments relative to others, leading to significant diffusion gradients on the scale of the compartment length. This suggests that the establishment of concentration gradients is an important driving force in actomyosin self-organization, as has been noted in other work [43,44]. In the initial transient phase, the mechanical energy changes appreciably as the filaments grow and are initially coupled to each other. Following this, however, the rate of $\Delta G_{\mathrm{mech}}^{+}$ is on average near zero. This indicates that, despite the process of mechanical stress generation through myosin filament activity and treadmilling, the resulting stress is dissipated through fast relaxation such that, on a slower time scale, the mechanical energy of the system does not change in a significant, persistent way.

**Figure 5.**
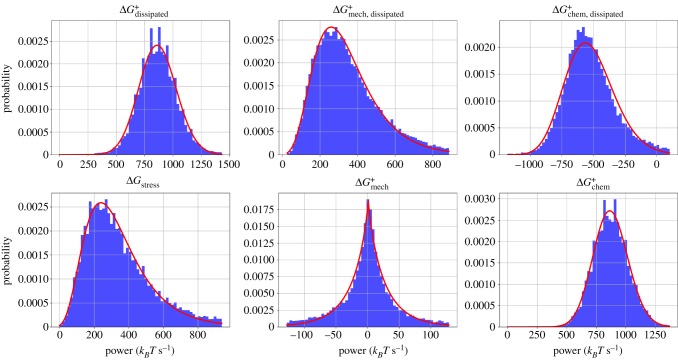
Histograms and fitted probability distribution functions for six tracked quantities. For each histogram, the full trajectory for each of 10 runs is combined into a single dataset. A lognormal distribution was used to fit the histograms of $\Delta G_{\mathrm{dissipated}}^{+}$ and $\Delta G_{\mathrm{chem}}^{+}$, a generalized normal distribution was used to fit $\Delta G_{\mathrm{mech}}^{+}$, and the rest were fitted with gamma distributions. All distributions are fitted using the SciPy package to determine shape, scale and location parameters [45]. Quantities were made positive or negative in order to produce the best fits. (Online version in colour.)

Aggregating each of the 2000 s of the 10 trajectories into a collective dataset for each condition of *C*_M_ and *C*_CL_, we next analysed the distribution of the instantaneous rates of $\Delta G_{\mathrm{dissipated}}^{+}$, $\Delta G_{\mathrm{mech},\;\mathrm{dissipated}}^{+}$, $\Delta G_{\mathrm{chem},\;\mathrm{dissipated}}^{+}$, $\Delta G_{\mathrm{stress}}^{+}$, $\Delta G_{\mathrm{mech}}^{+}$, and $\Delta G_{\mathrm{chem}}^{+}$. In figure 5, we plot histograms of these six quantities for the conditions *C*_CL_ = 1.0 μM, *C*_CL_ = 0.2 μM. In electronic supplementary material, figures S13 and S14, we show the same plots for other conditions.

Each distribution contained heavy tails which, for the purpose of fitting, were suppressed by excluding data lying outside 5 MADs of the median. No distributions were sufficient to cleanly fit the full set of data, so we focus here on the centre of the distribution and include a qualitative discussion of the heavy tails below. With the exception of $\Delta G_{\mathrm{mech}}^{+}$ which was fitted with a generalized normal distribution, each distribution exhibited significant skew and could be fitted reasonably well with a lognormal or a gamma distribution. At higher concentrations of myosin and cross-linkers, the histograms were less cleanly fitted by any standard distributions (electronic supplementary material, figure S14).

Lognormal distributions are fairly ubiquitous across different fields and systems [46]. For instance, it has been shown that the distributions of concentrations of species in a chemical reaction network are lognormal [47]. Gamma distributions are similarly common and often difficult to discriminate from lognormal distributions [48]. A speculative explanation for the gamma distribution of Δ*G*_stress_ is as follows: Δ*G*_stress_ can be viewed as resulting from a number of myosin filament steps that, according to the central limit theorem, is approximately normally distributed given a sufficiently long time between simulation snapshots *t*_snap_ (these stepping events are not truly independent and identically distributed, but to a first approximation we may assume they are). The main effect of each of these steps is to increase the harmonic stretching potential on the myosin filaments as well as on the actin filament cylinders by a roughly fixed stepping distance. Thus the increase in mechanical energy, Δ*G*_stress_, is approximately a quadratic function of the normally distributed number of myosin filament steps per *t*_snap_. As shown in electronic supplementary material, figure S15, the resulting distribution of Δ*G*_stress_ is well fitted by a gamma distribution and bears qualitative similarity to the histogram of Δ*G*_stress_ in figure 5. In figure 5, Δ*G*_stress_ and $\Delta G_{\mathrm{mech},\;\mathrm{dissipated}}^{+}$ have similar distributions, indicating that, for each MEDYAN cycle, almost all the stress accumulated following Step 1 is then immediately relaxed. The remainder goes into $\Delta G_{\mathrm{mech}}^{+}$, whose distribution is centred on zero with little skew. Furthermore, the distribution of $\Delta G_{\mathrm{mech},\;\mathrm{dissipated}}^{+}$ has particularly heavy tails, indicating infrequent yet large relaxation events. This tendency has some precedent in avalanche-prone systems, whose hallmarks are self-organized criticality, intermittency, and scale-invariance in their distribution of avalanche event sizes [49–52]. While we do not observe true scale-invariance in this set of simulations (i.e. power-laws cannot fit these distributions cleanly), it will be interesting to continue to explore actomyosin network dynamics in the framework of self-organized criticality [53].

### 3.2. More compact networks are more efficient

**Figure 6.**
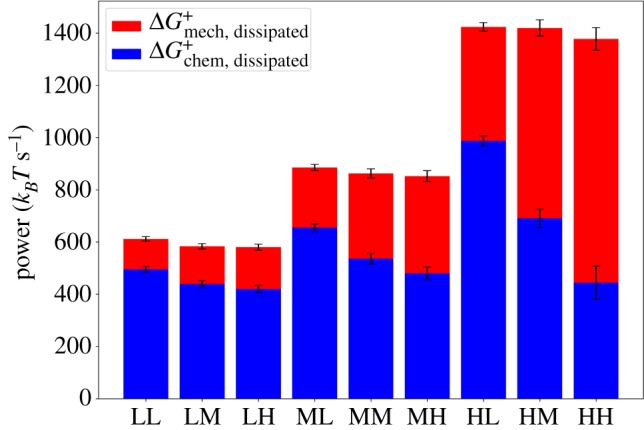
Bar plot representing the contributions of $\Delta G_{\mathrm{chem},\;\mathrm{dissipated}}^{+}$ and $\Delta G_{\mathrm{mech},\;\mathrm{dissipated}}^{+}$ to the total, $\Delta G_{\mathrm{dissipated}}^{+}$. The letters in the abscissa labels designate ‘low’, ‘medium’ and ‘high’. The first letter represents the concentration of myosin, *C*_M_ : `L′ = 0.1 μM, `M′ = 0.2 μM, `H′ = 0.4 μM, and the second letter represents the concentration of cross-linkers, *C*_CL_ : `L′ = 0.1 μM, `M′ = 1.0 μM, `H′ = 5.0 μM. The median of each quantity is taken over 10 runs of 2000 s, and error bars represent 1 MAD. (Online version in colour.)

We simultaneously varied concentrations of myosin filaments *C*_M_, and cross-linkers, *C*_CL_, which is known to produce a range of network architectures [34,54]. Using cross-linker concentrations of 0.1, 1.0 and 5.0 μM, and myosin concentrations of 0.1, 0.2 and 0.4 μM, we studied the effects on $\Delta G_{\mathrm{dissipated}}^{+}$, $\Delta G_{\mathrm{chem},\;\mathrm{dissipated}}^{+}$ and $\Delta G_{\mathrm{mech},\;\mathrm{dissipated}}^{+}$. The concentrations of actin subunits and filaments are the same as described above. For each of the 9 conditions, we ran 10 simulations of 2000 s. In figure 6, we display the median values of $\Delta G_{\mathrm{chem},\;\mathrm{dissipated}}^{+}$ and $\Delta G_{\mathrm{mech},\;\mathrm{dissipated}}^{+}$ along each trajectory and over each repeated trajectory for these conditions. The median is used here because of its insensitivity to outliers such as are found in the heavy-tailed distributions of these quantities [42].

We find that as *C*_M_ is increased, the median rates of $\Delta G_{\mathrm{dissipated}}^{+}$, $\Delta G_{\mathrm{chem},\;\mathrm{dissipated}}^{+}$ and $\Delta G_{\mathrm{mech},\;\mathrm{dissipated}}^{+}$ all tend to increase. Furthermore, the value of $\Delta G_{\mathrm{mech},\;\mathrm{dissipated}}^{+}$ relative to $\Delta G_{\mathrm{chem},\;\mathrm{dissipated}}^{+}$ increases. As *C*_CL_ is increased with *C*_M_ fixed, then $\Delta G_{\mathrm{chem},\;\mathrm{dissipated}}^{+}$ tends to decrease. Increased concentrations of myosin filaments obviously have a strong effect on the dissipation simply because they are the chief active agents in the system once treadmilling has reached its steady state. We can thus define a measure of efficiency for the present purpose:

3.1 $$\begin{array}{l} \eta =\frac{\Delta G_{\mathrm{stress}}}{\Delta G_{\mathrm{chem}}^{+}}=\frac{\Delta G_{\mathrm{mech},\;\mathrm{dissipated}}^{+}-\Delta G_{\mathrm{mech}}^{+}}{\Delta G_{\mathrm{dissipated}}^{+}-\Delta G_{\mathrm{mech}}^{+}}\\ \;\approx \frac{\Delta G_{\mathrm{mech},\;\mathrm{dissipated}}^{+}}{\Delta G_{\mathrm{dissipated}}^{+}}, \end{array}$$

where the approximation follows since, as illustrated in figure 2, the average of $\Delta G_{\mathrm{mech}}^{+}$ is close to zero. Thus it is evident that, perhaps surprisingly, as *C*_M_ increases, *η* increases: the more motors are in our system, the more efficiently can chemical energy be converted into mechanical stresses. Furthermore, as *C*_CL_ increases, *η* tends to increase because Δ*G*_chem, dissipated_ decreases relative to Δ*G*_mech, dissipated_. At higher levels of cross-linking, which introduce mechanical constraints on the filaments, the walking of myosin motor filaments will produce more stress compared to at low levels of cross-linking, when filament sliding can result from myosin filament walking, producing less stress.

We repeated the above experiments with the inclusion of mechanochemical feedback on the reaction rates controlling cross-linker unbinding and myosin filament unbinding and walking (electronic supplementary material, figures S16–S18). We observed, somewhat surprisingly, no qualitative differences compared to the results described for the case of no feedback; however, there were quantitative differences in the stress production and radius of gyration for certain conditions. These quantitative differences result from the fact that, for the concentrations *C*_M_ and *C*_CL_ used above, we did not observe significant collapse of the actomyosin network when mechanochemical feedback was included due to the stalling and catching of the myosin filaments. As a result, the networks were less densely cross-linked, and the efficiency was lower. The total dissipation rates are largely unaffected by the inclusion of mechanochemical feedback; it was primarily the degree to which chemical energy had been converted to stress that was different for certain conditions.

It is worthwhile to mention how these results are compared with *in vitro* studies of dissipation in actomyosin systems. While the computational approach described in this paper allows uniquely highly resolved and direct measurement of free energy changes, other experimental methods have produced qualitatively similar results to those obtained here. Rheological experiments have determined that a single cell’s response to compression is similar, in nature, to a muscle’s response to increasing load, suggesting that the actomyosin network underlies the cell’s mechanical responsiveness [31]. Furthermore, this responsiveness is modulated by blebbistatin, a myosin ATPase inhibitor, highlighting myosin’s role in negotiating how the network rearranges in response to the sustained stress. Using the metric of mechanical dissipation to measure the degree of structural rearrangements and release of stress, we confirm that these processes indeed sensitively depend on myosin activity, which we control here through its concentration. Additional rheological studies have probed the mechanical dissipation of actin cortices more directly, using the loss modulus as a readout. These have also indicated that inhibiting myosin activity reduces the mechanical dissipation of the system, causing it to be behave more elastically [30]. Finally, we mention a recent study that quantified mechanical dissipation of actin filaments using a novel experimental method [55]. By measuring the flow through a low-dimensional phase space defined by the amplitudes of the filament bending modes [56], they determine the entropy production of fluctuating actin filaments in different phases of contractility. They relate the entropy production to the degree of transverse bending of the filaments, as opposed to sarcomeric filament sliding, caused by myosin filament walking. Similarly, we here relate the hindrance of filament sliding due to cross-linker density to increased mechanical dissipation rates. Quantitative comparisons of these two approaches to mechanical dissipation measurement will be an interesting future direction. We note finally that a unique capability of quantifying dissipation using MEDYAN is the ability to simultaneously measure the energetics of chemical reactions in addition to the changes in the mechanical energy, which is not currently available using *in vitro* methods.

## 4. Discussion

We have introduced a methodology for tracking the energetics of chemical and mechanical events during a MEDYAN simulation, allowing us to probe the properties of actomyosin networks as dissipative active matter systems. The distinction between dissipation’s mechanical and chemical origins is natural in the context of MEDYAN’s iterative simulation procedure which carries out chemical stochastic simulation, mechanical deformation and mechanical relaxation at separate times. As explained in [34], this procedure exploits a separation of time scales between the characteristic mechanical relaxation times of actomyosin networks [57] compared to typical waiting time between reactions that introduce mechanical stresses, such as myosin filament walking [10] or filament growth [58]. Ultimately, the source of all dissipation is the chemical potential of ATP molecules driving treadmilling and myosin filament activity. This is reflected by the near equality of $\Delta G_{\mathrm{chem}}^{+}$ and $\Delta G_{\mathrm{dissipated}}^{+}$ in figure 2 and electronic supplementary material, figures S6 and S7. On a fast time scale (that of the characteristic mechanical relaxation time), however, the free energy of chemical reactions causes small force deformations of the network which are then quickly and almost fully relaxed. Thus, for a myosin filament stepping event, only a portion of the chemical free energy $\Delta G_{\mathrm{chem},\;\mathrm{dissipated}}^{+}$ is immediately dissipated as heat, with the rest going into temporarily increased mechanical energy of the actomyosin network, Δ*G*_stress_. The fast relaxation of this new mechanical stress constitutes what we refer to as mechanical dissipation, $\Delta G_{\mathrm{mech},\;\mathrm{dissipated}}^{+}$, and the small residual stress after this relaxation has balanced all net forces acting on the system results in a change of the mechanical energy of the system on a slow time scale, $\Delta G_{\mathrm{mech}}^{+}$.

**Figure 7.**
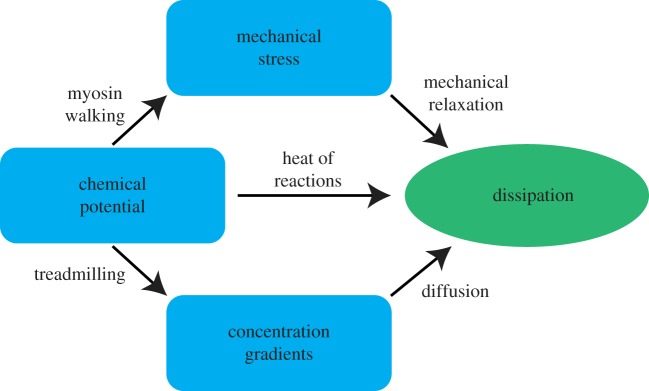
A simple schematic illustrating the flow of free energy in actomyosin network systems. Blue compartments represent forms of free energy storage, arrows represent the transfer of free energy from one form to another, and the green compartment indicates dissipation as ultimate destination of all free energy flows in this non-equilibrium system. Arrows are labelled with the mechanisms by which these free energy transductions from one form to another are achieved. In this depiction, the sizes of compartments and the widths of the arrows indicating the magnitudes of the represented quantities are not to scale. (Online version in colour.)

One interpretive framework, which is useful to understand the flow of free energy in actomyosin networks, is illustrated in figure 7. We can think of the different forms of free energy storage, including chemical potentials, mechanical stress, concentration gradients (which could also be considered as arising from chemical potential differences across compartments) and dissipated energy, as nodes on a directed graph, where edges represent transduction of energy from one form of storage to another. The weights of these edges represent the amount of free energy flowing through them. In this picture, we can describe the process of network percolation, which occurs at increasing concentrations of cross-linkers and myosin filaments, as widening the edge flowing from chemical potential into mechanical stress, while thinning the edge from chemical potential directly to dissipation. The edge weights corresponding to the establishment of concentration gradients and the resulting diffusive dissipation will not be affected dramatically by the onset of percolation except to the extent that percolated networks might lead to the formation of more bundles, and therefore more significant concentration gradients. At steady state, we have a stationary current on the graph, fuelled by the chemical potential of the assumed limitless supply of ATP. Of course, at this level of description, we have coarse-grained away the details of the specific chemical reaction networks and mechanical potentials which constitute the system; however by doing so, we gain a clearer understanding of how percolation of the network alters the flows of free energy.

The new capabilities of MEDYAN allowing detailed energetic computations should provide a way to address outstanding issues in the fields of active matter systems and of cell mechanics. In this study, we observe dissipation rates to stay at a low fluctuating steady-state value following a high initial transient phase. This does not necessarily contradict the dissipation-driven adaptation hypothesis, which is a statement about the history of a system's dissipation rather than its eventual possible outcomes [25]. We also do not observe slow reorganization of the actomyosin networks into different higher order structures such as bundles (for the concentrations of components used here we observe disordered networks only), and as a result we cannot rule out that such reorganizations correspond to marked changes in the dissipation rates. A dedicated test of the hypothesis of dissipative-driven adaptation should be straightforward with this methodology. For instance, a simulated gliding assay, which has been shown *in vitro* to lead to diverse dynamical patterns [20], may indicate that these emergent patterns correspond to an optimization of the chemical dissipation from myosin filament walking, as argued in [25]. In the context of cell migration, studies investigating the mechanically dissipative activity of myosin filaments are also feasible if one incorporates in simulation an external substrate against which the actomyosin network pulls. In this type of study, it should be straightforward to determine how the amount of stress which is sustained against the substrate is altered by myosin activity given the energetics calculations in the methodology described in this paper. It is also feasible to do simulated measurements of the loss modulus by compressing the actomyosin network at different frequencies. It should then be possible to directly observe the degree to which the dissipation of elastically stored energy is attributable to myosin walking. In fact, this last research question has already been investigated to some extent using an alternate computational model to the one presented here [59]. We hope some of these potential future experiments will shed light on outstanding questions in the studies of actomyosin networks.

## Supplementary Material

Supplementary Information

## Footnotes

1 In our terminology, we will distinguish between ‘cross-linking proteins’, which will include both active (e.g. myosin filaments) and passive (e.g. *α*-actinin and fascin) proteins that bind to adjacent actin filaments, and ‘cross-linkers’, which refer exclusively to passive cross-linking proteins.

2 For certain statistical aggregations in this paper, we use the MAD since it is the estimate of scale most robust against outliers, having a breakdown point of 50%. However, we use the unscaled version of the MAD and, as a result, do not claim that this statistic is a consistent estimator of the standard deviation [42]. The distributions of most of the quantities of interest are too pathological to allow for a straightforward choice of scaling factor, so we simply use the unscaled version, defined as MAD = median_*i*_|*x*_*i*_ − median_*j*_*x*_*j*_|, where *x*_*i*_ are elements in the dataset.
