# Supplementary material for: Quantifying dissipation in actomyosin networks
Source: Interface Focus. 2019 Apr 19;9(3):20180078. doi: 10.1098/rsfs.2018.0078 (PMC6501337; doi:10.1098/rsfs.2018.0078)
Supplement: Supplementary Information [file rsfs20180078supp1.pdf]

Supplementary Information for "Quantifying Dissipation in Actomyosin Networks"

Carlos Floyd[1], Garegin Papoian[1,2,3,†], and Christopher Jarzynski[1,2,3,4,*]

[1]Biophysics Program, University of Maryland, College Park, MD 20742 USA
[3]Institute for Physical Science and Technology, University of Maryland, College Park, MD 20742 USA
[2]Department of Chemistry and Biochemistry, University of Maryland, College Park, MD 20742 USA
[4]Department of Physics, University of Maryland, College Park, MD 20742 USA
[*]email: cjarzyns@umd.edu
[†]email: gpapoian@umd.edu

February 24, 2019

1 Supplementary Methods

1.1 ΔG of Chemical Reactions

For a reaction of the general form

$$\nu_1 X_1 + \nu_2 X_2 + \ldots \rightleftharpoons \upsilon_1 Y_1 + \upsilon_2 Y_2 + \ldots \tag{1}$$

we define X_i as the reactant species, Y_i as the product species, and ν_i and υ_j as their stoichiometric coefficients. The "stoichiometric difference" is defined as

$$\sigma = \sum_{j \in P} \upsilon_j - \sum_{i \in R} \nu_i \tag{2}$$

where P is the set of products and R is the set of reactants. We further define the conversion factor between the copy number of species i, N_i, and its concentration C_i,

$$\Theta = N_{\mathrm{Av}} V \tag{3}$$

where N_{Av} is Avogadro's number, V is the volume of the compartment where the reaction occurs, and we have $N_i = C_i \Theta$. Next we define the following quantity, reminiscent of the reaction quotient:

$$\widetilde{Q} = \prod_{i \in R} \frac{(N_i - \nu_i)^{N_i - \nu_i}}{N_i^{N_i}} \prod_{j \in P} \frac{(N_j + \upsilon_j)^{N_j + \upsilon_j}}{N_j^{N_j}} \tag{4}$$

where R is the set of reactant species and P is the set of product species. Lastly, defining ΔG^0 as the standard state change in Gibbs free energy, we arrive at the following expression for the change in Gibbs free energy as a function of the instantaneous vector of species copy number $\mathbf{N}$:

$$\Delta G(\mathbf{N}) = \Delta G^0 - \sigma k_B T \log \Theta - \sigma k_B T + k_B T \log \widetilde{Q}. \tag{5}$$

Although this equation might appear unusual, upon making some approximations leveraging the relative size of the stoichiometric coefficients and the species copy numbers it can be shown to reduce to the familiar textbook expression

$$\Delta G(\mathbf{C}) = \Delta G^0 + k_B T \log Q \tag{6}$$

where

$$Q = \prod_{i \in R} C_i^{-\nu_i} \prod_{j \in P} C_j^{\upsilon_j} \tag{7}$$

is the reaction quotient of the species concentrations C_i, and $\mathbf{C}$ is the vector of these concentrations. In the context of a compartment-based reaction diffusion scheme, the species copy numbers used in Equation 5 are specific to the compartment in which the reaction was drawn. We prefer to use Equation 5 in simulation because small deviations from this nearly exact expression can actually lead to significant systematic bias of the change in Gibbs free energy resulting from certain reactions. This is especially true for reactions that are very frequent, such as the diffusion reaction between adjacent compartments. ΔG for diffusion reactions, in which a molecule jumps from a compartment where its concentration is $N_{i,A}$ to a compartment where its concentration is $N_{i,B}$, is calculated using an expression similar to Equation 5:

$$\Delta G = k_B T \log \frac{(N_{i,A} - 1)^{(N_{i,A} - 1)}}{N_{i,A}^{N_{i,A}}} \frac{(N_{i,B} + 1)^{(N_{i,B} + 1)}}{N_{i,B}^{N_{i,B}}}. \tag{8}$$

Similarly this expression reduces to the familiar formula

$$\Delta G = k_B T \log \frac{N_{i,B}}{N_{i,A}} \tag{9}$$

upon leveraging the sizes of $N_{i,A}$ and $N_{i,B}$ relative to 1. The derivation of the above results can be found in an accompanying paper [1].

For some of the reactions in the CT model, Equation 6 can be directly applied and straightforwardly cast into the form of Equation 5 to give more exact results. However other reactions including (de)polymerization, myosin filament walking, myosin filament (un)binding, and cross-linker (un)binding, require some additional treatment.

For (de)polymerization reactions, care should be taken in defining the concentrations of the reactants and products since those molecules include heteropolymers; we would like to avoid requiring that a specific sequence of distinct subunits constitutes a unique chemical species. This is possible to do because the polymerization of actin subunits is independent of the chemical identity of the subunit at the tips of the polymer, and is therefore independent of the chemical identity of any of the polymerized subunits [2]. Then, polymerization potentially only depends on the polymer length, such that a general reaction for reversible polymerization can be written as

$$(m)_n + m \longleftrightarrow (m)_{n+1} \tag{10}$$

where $(m)_n$ is a polymer with degree of polymerization n and m is a subunit. For this reaction Equation 6 reads

$$\Delta G = \Delta G^0 + kT \log \frac{C_{(m)_{n+1}}}{C_{(m)_n} C_m}. \tag{11}$$

Following [3], we make the simplifying assumption that a polymer's chemical reactivity does not depend on its degree of polymerization, provided that the polymer is sufficiently long and cooperative effects do not apply. With this, Equation 11 reduces to

$$\Delta G = \Delta G^0 + kT \log \frac{1}{C_m}. \tag{12}$$

This assumption agrees more or less with intuition: the polymerizing subunit does not really "see" the degree of polymerization of the polymer, which is reflected by constant rates of polymerization for polymers of varying lengths.

Cross-linking proteins are incorporated in MEDYAN as diffusing species that bind and mechanosensitively unbind to pairs of actin filaments, mechanically coupling them. We treat the change in free energy of the (un)binding reactions analogously to the (de)polymerization reactions. Ignoring the chemical identities of the heteropolymer subunits, a general cross-linking protein (un)binding reaction can be written as

$$(m)_i + (m)_j + L \longleftrightarrow (m)_i L (m)_j \tag{13}$$

where L is a cross-linking protein and $(m)_i$ is a polymer with degree of polymerization i. According to Equation 6, the change in Gibbs free energy upon this reaction occurring to the right is

$$\Delta G = \Delta G^0 + kT \log \frac{C_{(m)_i L (m)_j}}{C_{(m)_i} C_{(m)_j} C_L}. \tag{14}$$

Similarly to our assumption that the chemical reactivity of polymers is independent of degree of polymerization for sufficiently long polymers, we assume here that the binding affinity of cross-linking proteins is independent of the degree of polymerization and of the number of cross-linking proteins already bound to the pair of filaments, allowing us to simplify Equation 14 to

$$\Delta G = \Delta G^0 + kT \log \frac{1}{C_L}. \tag{15}$$

The kinetics of myosin filament walking in MEDYAN are based on the Parallel Cluster Model (PCM) [4]. Taking the rate constants of individual myosin head (un)binding reactions as inputs, and accounting for the statistical distribution of the number of bound heads as well

as dependence on the force exerted on the filament, the results of the PCM allow us to write kinetic parameters describing the entire myosin filament, including filament (un)binding and walking rates. In the MEDYAN implementation, each step of a myosin filament can represent several steps of the constituent myosin heads, where each head step represents the completion of a single myosin head cross-bridge cycle [5, 6]. The head steps have a fixed length, d_{step}, set by experimental measurements of the myosin isoform of interest. The length of the filament steps, d_{total}, is determined from the following MEDYAN parameters: the equilibrium length L_{cyl} of the cylinders that comprise the coarse-grained representation of actin filaments, and the number of binding sites per cylinder N_{bs}, giving $d_{\text{total}} = L_{\text{cyl}}/N_{\text{bs}}$. The binding sites represent the discrete locations on the cylinders which can be occupied by cross-linkers or myosin filaments. To account for the discrepancy between d_{step} and d_{total}, we multiply the filament walking rate by the ratio

$$s = \frac{d_{\text{step}}}{d_{\text{total}}}. \tag{16}$$

When a filament step occurs in MEDYAN, it thus represents the completion of s^{-1} myosin head cross-bridge cycles, each of which has the effect of converting one solvated ATP molecule into solvated Pi and ADP. The Gibbs free energy the filament walking reaction is then

$$\Delta G = s^{-1} \left(\Delta G^0 + k_B T \log \frac{C_{ADP} C_{Pi}}{C_{ATP}} \right). \tag{17}$$

where ΔG^0 refers to the standard change in Gibbs free energy for hydrolysis of ATP. This expression for ΔG could be further multiplied by a parameter ζ representing the coupling of the ATP hydrolysis cycle to the forward step of the myosin head; here we follow the assumption of tight coupling, i.e. $\zeta = 1$ [6, 7].

The mechanochemical updating of force-sensitive reaction rates $k_\pm$ alters the equilibrium constant via $K_{\text{eq}} = k_-/k_+$, and therefore the change in free energy ΔG corresponding to that reaction via $\Delta G^0 = k_B T \log K_{\text{eq}}$. For a reaction whose rates have been updated due to some applied force F, it can be shown that the new value of ΔG^0 is approximately given by

$$\Delta G^0(F) = \Delta G^0|_{F=0} + g(F) \tag{18}$$

where $G^0|_{F=0}$ is the original (i.e. zero-force) value of ΔG^0 and $g(F)$ is equal to the increase mechanical energy due to the applied force [6, 8]. In this modeling, the extra energy $g(F)$ is counted as a part of $\Delta G_{\text{mech, dissipated}}$, not $\Delta G_{\text{chem, dissipated}}$. So when, for example, a cross-linking protein unbinds under tension F, the zero-force value $\Delta G^0|_{F=0}$ is used when computing the change in free energy for that reaction, and when mechanical equilibration next occurs, the released stretching energy $g(F)$ is included in the calculation of $\Delta G_{\text{mech, dissipated}}$.

1.2 Parameterization

Parameterization of the CT model for the purpose of tracking free energy changes during simulation trajectories consists of choosing values of the rate constants (kinetic parameters) and of ΔG^0 (thermodynamic parameters) for all reactions in the model. Wherever possible, values from the literature are used. Experimental measurements have determined rate constants for every reaction, however for some reactions the value of ΔG^0 hasn't been reliably measured, to the best of our knowledge. Below, we describe a technique to solve for these unknown values.

For reversible reactions, where the forward and reverse rate constants k_+ and k_- are known, such as (de)polymerization and (un)binding of cross-linkers, ΔG^0 can be found from

$$\Delta G^0 = k_B T \log K_{\text{eq}} = k_B T \log \frac{k_-}{k_+} \tag{19}$$

Literature values for the equilibrium constants or of ΔG^0 are used for irreversible reactions, for which k_- is often too small to determine from direct measurement. Irreversible reactions in this system include all reactions except for (de)polymerization reactions. For reactions for which literature values of ΔG^0 are unavailable, it is possible to solve for ΔG^0 values based on a self-consistency condition [9, 10]: the sum of the ΔG^0 values around a closed loop of reactions in which the number of molecules has not experienced a net change must be zero since the free energy is a state function (equivalently, by Equation 19, the product of equilibrium constants around any such loop must be equal to one). Writing several such closed loops of reactions leads to a system of equations that can be solved for the unknown variables. Not all possible loops result in independent equations, but we were able to determine the values of two unknown parameters using the loops illustrated in SI Figure 1.

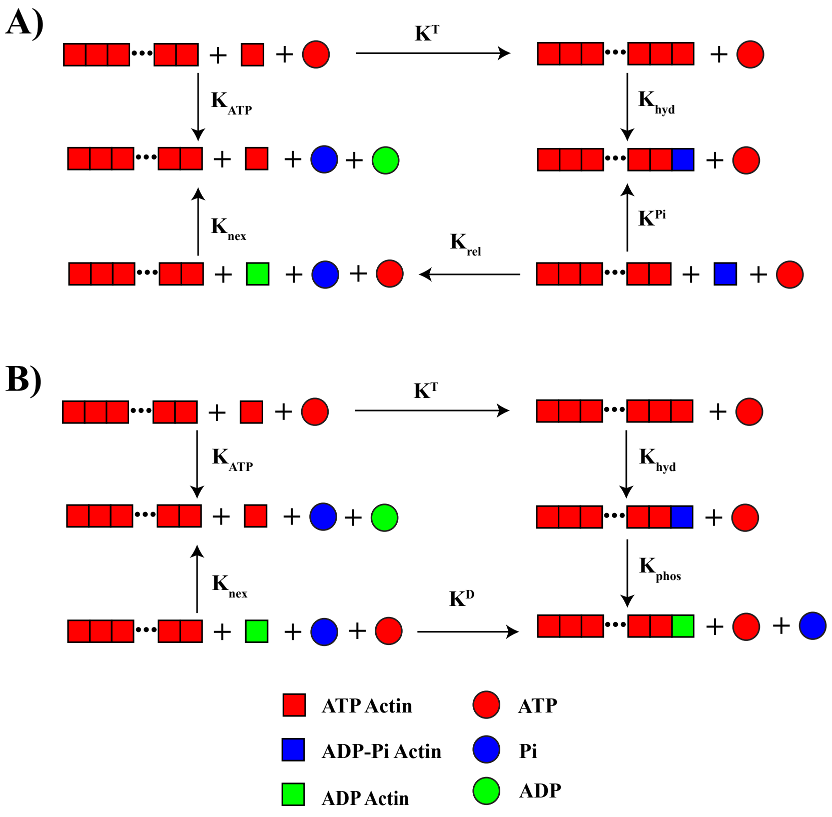

Figure 1: Diagrams representing sequences of reactions, with the involved species drawn between each reaction, resulting in independent relations between the equilibrium constants. The meaning of these equilibrium constants is provided in the main text. Loops are assumed to proceed in the clockwise direction, and arrows that point opposite to this direction indicate that that reaction is occurring "backwards", i.e. from products to reactants. Polymers are shown as connected chains of subunits, while the "+" sign indicates different solvated species.

The loops in SI Figure 1 imply the following independent system of equations:

$$K^T K_{\text{hyd}} K_{\text{rel}} K_{\text{nex}} = K^{Pi} K_{\text{ATP}} \tag{20}$$

$$K^T K_{\text{hyd}} K_{\text{phos}} K_{\text{nex}} = K^D K_{\text{ATP}} \tag{21}$$

where K_{hyd} represents the hydrolysis of ATP by F^T, K_{phos} represents the release of Pi by F^{Pi}, K_{nex} represents nucleotide exchange converting G^D to G^T, K_{ATP} represents the hydrolysis of ATP in solution producing ADP and Pi, and K_{rel} represents the release of phosphate by G^{Pi}.

Values from the literature [11–13] can be used to determine 6 of the 8 variables in Equations 20 and 21, which thus represent two equations in two unknowns: K_{rel} and K_{nex}. The resulting parameters are listed in Table 1.

Note that it is possible to draw loops such as those in SI Figure 1 that would imply that the equilibrium constants for polymerization and depolymerization of, for example G^T, should be the same at the plus and minus ends of the filaments. This condition is not borne out by the experimental values of these equilibrium constants, and this discrepancy is a recognized outstanding problem [11]. Here, we use the literature values for these equilibrium constants and employ the reaction loop method only to determine the parameters K_{rel} and K_{nex}.

For reversible binding of myosin filaments, results from the PCM are used to describe binding and unbinding rates, and therefore K_{eq} [4, 5]. The filament binding rate is given as

$$k_{\text{fil, bind}} = N_t k_{\text{head, bind}}, \tag{22}$$

and the unbinding rate is

$$k_{\text{fil,unbind}} = N_t k_{\text{head, bind}} \left[\left(\frac{k_{\text{head, bind}} + k_{\text{head, unbind}}}{k_{\text{head, unbind}}} \right)^{N_t} - 1 \right]^{-1}, \tag{23}$$

where $k_{\text{head, bind}}$ and $k_{\text{head, unbind}}$ describe the binding kinetics of a single myosin head, and N_t is the number of heads in the filament [4]. The resulting expression for ΔG^0 is

$$\Delta G^0 = kT \frac{k_{\text{fil, unbind}}}{k_{\text{fil, bind}}} = -kT \log \left[\left(\frac{k_{\text{head, bind}} + k_{\text{head, unbind}}}{k_{\text{head, unbind}}} \right)^{N_t} - 1 \right]. \tag{24}$$

We assume that we have chemostatted concentrations of ATP, ADP, and Pi, which we account for implicitly via the effect of these concentrations on the kinetic and thermodynamic parameters of certain reactions. Thus these species are not explicitly tracked. The concentrations of these species affect the change in Gibbs free energy associated with the following reactions:

- myosin filament walking
- nucleotide exchange ($G^D \to G^T$)
- phosphate release by F and G-actin ($F^{Pi} \to F^D$, $G^{Pi} \to G^D$)

The effect of the concentrations of ATP, ADP, and Pi for the these reactions is to simply change the reaction quotient Q which changes ΔG via $\Delta G = \Delta G^0 + k_B T \log Q$. For instance the nucleotide exchange reaction can be written explicitly as

$$ATP + G^D \to ADP + G^T \tag{25}$$

and the change in Gibbs free energy is

$$\Delta G = \Delta G^0 + k_B T \log \left(\frac{C_{ADP} C_{G^T}}{C_{ATP} C_{G^D}} \right). \tag{26}$$

To treat the concentrations of ATP and ADP implicitly, we rewrite the reaction as

$$G^D \to G^T \tag{27}$$

for which the change in Gibbs free energy is

$$\Delta G = \Delta G^{0\prime} + k_B T \log \left(\frac{C_{G^T}}{C_{G^D}} \right), \tag{28}$$

where

$$\Delta G^{0\prime} = \Delta G^0 + k_B T \log\left(\frac{C_{ADP}}{C_{ATP}}\right). \tag{29}$$

A similar approach is taken for the other reactions mentioned above.

The concentration of just ATP (since neither ADP or Pi appear implicitly as reactants in any of the reactions of the CT model) affects the kinetics of the following reactions:

- myosin filament walking
- nucleotide exchange

To understand the effect of C_{ATP} on the myosin filament walking rate, we employ the five state cross-bridge model of a single myosin head described in [5]. In that model unbinding of a head from the filament substrate occurs via two pathways: a slip path, with rate k_{35}, and a catch path, with an effective rate k_{345}. The catch path is a two-step reaction: the release of ADP with rate k_{34}, followed by the unbinding of the filament head and binding of ATP with rate $k_{45} = k_T C_{ATP}$. The effective rate constant of the approximate one-step representation of this reaction is

$$k_{345} = \frac{k_{34} k_T C_{ATP}}{k_{34} + k_T C_{ATP}}. \tag{30}$$

Unless C_{ATP} is very low, this reaction rate is limited by k_{34}. Because the head can unbind by the catch or slip pathway, the rate for unbinding is

$$k_{\text{head, unbind}} = k_{35} + \frac{k_{34} k_T C_{ATP}}{k_{34} + k_T C_{ATP}} \tag{31}$$

The slip path should also be dependent on ATP concentration, since in state 5 the head is ATP-bound, however we follow the authors of [5] in neglecting this dependence since the slip path only becomes active under large load.

The rate of the nucleotide exchange reaction also depends on C_{ATP} and also occurs in two steps. The full reaction, with ATP and ADP explicitly included, is

$$G^D + ATP \rightarrow G^* + ATP + ADP \rightarrow G^T + ADP \tag{32}$$

where G^* represents actin with no bound nucleotide. Following [2], this reaction is approximated as a one-step irreversible reaction with ATP and ADP included explicitly, Equation 27. We can write the rate k_{nex} of this approximate reaction as:

$$k_{\text{nex}} = \frac{k_{D\to *} k_{*\to T} C_{ATP}}{k_{D\to *} + k_{*\to T} C_{ATP}} \approx k_{D\to *} \tag{33}$$

where $k_{D\to *}$ is the dissociation rate of ADP, $k_{*\to T}$ is the second order rate constant of ATP association, and the approximation holds except at low concentrations of ATP when ADP dissociation is no longer the rate-limiting step. The values of $k_{*\to T}$ and $k_{D\to *}$ are presented and discussed in [14–16].

Reaction	Rate Constant	ΔG^0 (k_BT)	Reference
G^T poly at plus end	11.6 $(\mu Ms)^{-1}$	-2.12 [a]	[2]
G^T depoly at plus end	1.4 s^{-1}	2.12 [a]	[2]
G^T poly at minus end	1.3 $(\mu Ms)^{-1}$	-0.51 [a]	[2]
G^T depoly at minus end	0.8 s^{-1}	0.51 [a]	[2]
G^{Pi} poly at plus end	3.4 $(\mu Ms)^{-1}$	-2.81 [a]	[11]
G^{Pi} depoly at plus end	0.2 s^{-1}	2.81 [a]	[11]
G^{Pi} poly at minus end	0.11 $(\mu Ms)^{-1}$	-1.75 [a]	[11]
G^{Pi} depoly at minus end	0.02 s^{-1}	1.75 [a]	[11]
G^D poly at plus end	2.9 $(\mu Ms)^{-1}$	0.59 [a]	[2]
G^D depoly at plus end	5.4 s^{-1}	-0.59 [a]	[2]
G^D poly at minus end	0.09 $(\mu Ms)^{-1}$	1.03 [a]	[2]
G^D depoly at minus end	0.25 s^{-1}	-1.03 [a]	[2]
Pi release by F-actin	0.002 s^{-1}	-7.31 [b,e]	[2, 11]
ATP hydrolysis by F-actin	0.3 s^{-1}	-10.0	[2, 12]
Pi release by G-actin	5 s^{-1}	-10.77 [c,e]	-
Nucleotide exchange	0.01 s^{-1} [e]	-6.76 [c,e]	[2]
Cross-linker binding	0.7 $(\mu Ms)^{-1}$	-0.85 [a]	[17]
Cross-linker unbinding	0.3 s^{-1}	0.85 [a]	[17]
Myosin head binding	0.2 s^{-1} [d]	- [d]	[17]
Myosin head unbinding	1.708 s^{-1} [d]	- [d]	[17]
Myosin filament walking	- [f]	-14.5 [e]	[18]

Table 1: Kinetics and thermodynamic parameters describing reactions in the CT model as well as cross-linker and myosin filament (un)binding and myosin filament walking.

a - Values of ΔG^0 determined via Equation 19.
b - ΔG^0 determined from K_{eq} given in [11].
c - ΔG^0 determined using constraints as described above.
d - Parameters describing the myosin filament obtained via Equations 22, 23, 24.
e - Depends implicitly on C_{ATP}, C_{ADP}, and C_{Pi}; given value applies to standard state.
f - Calculated in simulation using results of PCM, see [17].

In all the studies in this paper, implicit nucleotide concentrations are taken to be C_{ATP} = 8 mM, C_{ADP} = 7 μM, and C_{Pi} = 1 mM, corresponding roughly to the amounts found in human muscle after exercise [18]. Other parameters of the system, including mechanical constants, diffusion rates, screening lengths, and boundary cutoffs have been set to the same values given and discussed in [17]. Cylinder equilibrium lengths L_{cyl} are chosen as 27 nm with 4 binding sites per cylinder for myosin filaments and 1 binding site per cylinder for cross-linkers, giving approximately physiological values for stepping distances of myosin motor filaments and spacing along actin filaments of bound α-actinin. We note that the form of the mechanochemical models has been changed from those used in [17]; the modeling used here is current as of MEDYAN v3.2, and we refer readers to the documentation at http://www.medyan.org/ for further details.

1.3 Mean-Field Model of Treadmilling Dissipation

To validate the methods for quantifying dissipation using MEDYAN against a simpler representation of actin filament treadmilling, we developed a mean-field description of the dissipation resulting from chemical reactions in the CT model. Mean-field models of the trajectory of the vector of concentrations of species, $\mathbf{C}(t)$, have been formulated previously as an 11-dimensional system of ordinary differential equations (ODEs) ([2]), and in the CT model as a 5-dimensional

system of ODEs ([19]). These models describe the polymerization of a concentration N_{fil} of actin filaments in a pool of actin subunits of total concentration M. The subunit species tracked by these models are distinguished by their polymerization state and by the hydrolysis states of the nucleotide to which they are bound. The meaning of "mean-field" in this context is the assumption that reacting species are well-mixed over the entire system volume, therefore obeying mass-action kinetics and deterministic dynamics which can be represented by ODEs. For an instantaneous value of $\mathbf{C}$, we define a function $D_\Lambda(\mathbf{C})$ representing the instantaneous rate of dissipation due to a set of reactions Λ. Thus a solution of a mean-field $\mathbf{C}(t) = \mathbf{N}(t)/\Theta$, allows us to construct the trajectory of the dissipation rate, $D_\Lambda(\mathbf{C}(t))$. This function cannot capture the dissipation due to the activity of myosin filaments or cross-linkers, or from relaxation of mechanical stress, because these aspects are not included in the these mean-field models describing filament treadmilling. The benefit of such a mean-field model is that one can perform systematic variation of parameters with limited computational demands, and we use it here to study the effect of the parameters N_{fil} and M on the dissipation due to filament treadmilling.

The function $D_\Lambda(\mathbf{C}(t))$ can be written as a sum over the reactions $\lambda \in \Lambda$ of the instantaneous rate of change of the solution's Gibbs free energy due to that reaction:

$$D_\Lambda(\mathbf{C}(t)) = \sum_{\lambda\in\Lambda} \Delta G_\lambda(\mathbf{C}(t)) r_\lambda(\mathbf{C}(t)). \tag{34}$$

The expressions for $\Delta G_\lambda(\mathbf{C}(t))$ for different reactions are described above. The instantaneous rate of reaction λ, $r_\lambda(\mathbf{C}(t))$, is written as usual for mass-action kinetics as

$$r_\lambda(\mathbf{C}(t)) = \Theta k_\lambda \prod_{i\in R} C_i^{\nu_i} \tag{35}$$

where R is the set of reactant species for reaction λ, and where we have included the conversion factor Θ to convert $r_\lambda(\mathbf{C}(t))$ to units of s^{-1}.

To facilitate the study of dissipation due to filament treadmilling, we define a set of reactions Λ in the CT model which constitutes the dominant cycle a subunit undergoes in the treadmilling process: a) polymerization of G^T to the plus end, b) hydrolysis of ATP by F^T, c) release of Pi by F^{Pi}, d) depolymerization of G^D from the minus end, and e) nucleotide exchange converting G^D to G^T. We refer to this set of 5 reactions as the main treadmilling pathway (MTP). Alternative sequences of reactions whose net effect is similarly the conversion of one molecule of ATP to ADP and Pi are considered as of secondary importance and not included in this analysis.

This mean-field description of entropy production can be compared to the description of entropy production rates for chemically reactive systems that emerged from the Brussels school of thermodynamics. [20, 21]. The results of that school include the minimum entropy-production principle applicable in the linear regime [22], and the general evolution criterion applicable even in the the non-linear regime [23]. Their formalism typically considers the total entropy production rate as a volume integral over the local entropy production rate density, which is itself written as a sum over fluxes multiplied by their corresponding thermodynamics forces defined at each point of the system. This sum is decomposed into terms representing diffusion and terms representing chemical reactions, and the terms representing the chemical reactions are written such that the fluxes reflect the net reaction rate at that point in the system. In contrast, our mean-field description neglects concentration gradients and resulting diffusion fluxes, as we assume a homogeneous distribution of the chemical species. Equation 34 then represents only the chemical contribution to the entropy production, as a sum over the rates and affinities of the reactions in the system, implicitly integrating over the homogeneous system volume. We treat the forward and reverse direction for some chemical reaction as separate terms in Equation 34, so these rates cannot be considered fluxes which would include the reverse rate as well. This allows for more general sets of reactions which might include effectively irreversible

processes for which the reverse rate is negligible. However the set of reactions Λ could be chosen to include a reverse reaction for each forward reaction, with the result that these pairs of terms represent fluxes along the reversible reaction pathway. The parsimonious reaction set MTP is chosen not to fully describe the rate of entropy production in the system, but to allow easy visualization of the main contributions to the entropy production. We do not pursue the connection of our treatment to the formalism of the Brussels school further here, but we lastly note that our results are compatible with their minimum entropy-production principle, as shown in SI Figure 2.

We first verified that the mean-field model of MTP dissipation agreed with results from MEDYAN simulations, to illustrate consistency between these approaches. In SI Figure 2 we display the close match between the trajectory of MTP dissipation over a 2000 s run from these two approaches. Note that, to allow direct comparison, only the changes of Gibbs free energy resulting from reactions in the MTP set are visualized for both approaches here, i.e. the contribution from diffusion and other non-MTP reactions in the MEDYAN simulation are not visualized. We also turned off in MEDYAN the force-sensitive decrease in polymerization rate when the filament tips push against the simulation hard-wall boundaries, since this effect is not captured in the mean-field modeling [24]. We used a simulation volume of 1 μm^3 and initial conditions of equal amounts (10 μM each) of G^T and G^D actin in a 0.08 μM pool of seed filaments containing F^T. The dissipation rate decreases nearly monotonically, attaining a minimal steady-state value after tens of seconds. In SI Figure 2 we also display the individual contributions to the sum in Equation 34. Initial polymerization of G^T to the plus end constitutes the majority of the initial dissipation. As this polymerization process slows after about 1 second, the hydrolysis of ATP by the now relatively abundant F^T becomes the dominant contribution. As hydrolysis then slows after about 10 seconds, the total dissipation rate reaches a steady-state value of roughly 80 k_BT/s. In SI Figure 2 we also plot the mean-field prediction of the trajectory of the reacting species' concentrations.

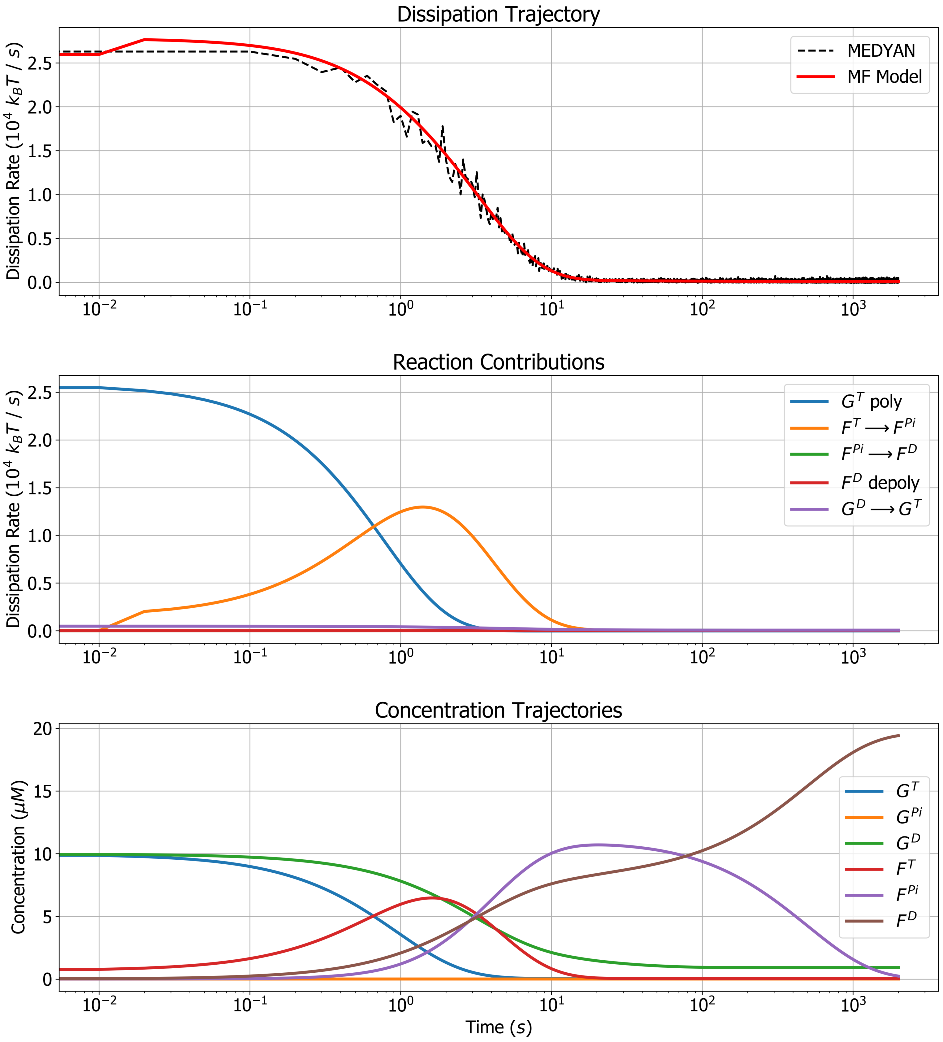

Figure 2: Results of the mean-field modeling of MTP dissipation. *Top*: Comparison of $D_{\mathrm{MTP}}(\mathbf{C}(t))$ calculated using the mean-field model (with time increments of 0.01 s), with ΔG_{chem} rates measured during MEDYAN simulation (with time increments of 0.1 s). *Middle*: Plot of the contributions of each reaction in the MTP to the total dissipation. The items in the legend represent reactions in the MTP, which are described in the main text. *Bottom*: Plot of the trajectories of the reacting species concentrations. The notation for each species is described in the main text.

We next simultaneously varied the total concentration of actin M and the concentration of actin filaments N_{fil}, and determined the total dissipation integrated along each trajectory as well as the steady-state dissipation rate. As M was varied, we held the initial concentration of each species proportionally the same: 49 % G^T, 49 % G^D, and 2 % F^T. As shown in SI Figure 3, the integrated dissipation over 2000 s was observed to increase monotonically with both M and N_{fil}. Quantitatively, the integrated dissipation depends on the choice of initial proportions,

however we found that the shape of the dependence on N_{fil} and M is largely independent of initial proportions (data not shown). Total dissipation increases with M simply because more actin is available to hydrolyze ATP during the approach to steady-state. For large amounts of actin, increasing N_{fil}, the number concentration of filaments, allows increased polymerization of G^T, which constitutes a large contribution to total dissipation during the early stages of the trajectory. Increasing N_{fil} also shifts the steady-state concentration of G^T downward (SI Figure 4), implying that more G^T has been polymerized during the approach to steady-state. A loose analogy can be made of a one lane road compared to a multi-lane highway during heavy traffic to describe this situation. As N_{fil} is increased with M fixed, the steady-state dissipation rate increases concavely, as shown in SI Figure 3. The steady-state concentration of F^T also increases concavely (SI Figure 4), representing higher rates of ATP hydrolysis. The contributions of each reaction to the total dissipation rate at steady-state as N_{fil} is varied is illustrated in SI Figure 5. The lack of dependence of the steady-state dissipation rate on M can be explained by the fact that increasing M increases the steady-state concentration of only F^D, not of any other species [19]. In other words, all extra actin accumulates in the form F^D as M is increased. This species is essentially inert, since the depolymerization rate of F^D is controlled by the concentration of filaments N_{fil}. Thus the steady-state dissipation has no dependence on the total amount of actin. Furthermore, it has no dependence on the initial concentrations, since it is known that the steady-state vector of concentrations does not depend on initial conditions [19].

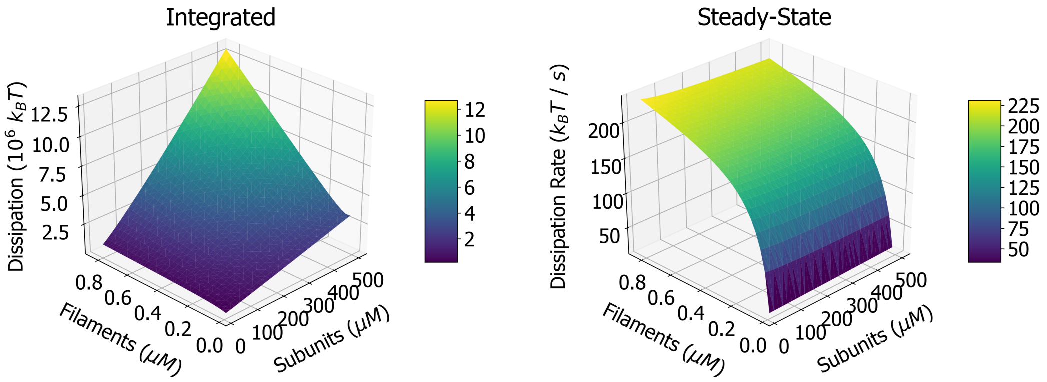

Figure 3: *Left*: The total dissipation integrated over 2000 s trajectories as M and N_{fil} are varied. *Right*: The steady-state dissipation rate over the same range of M and N_{fil}.

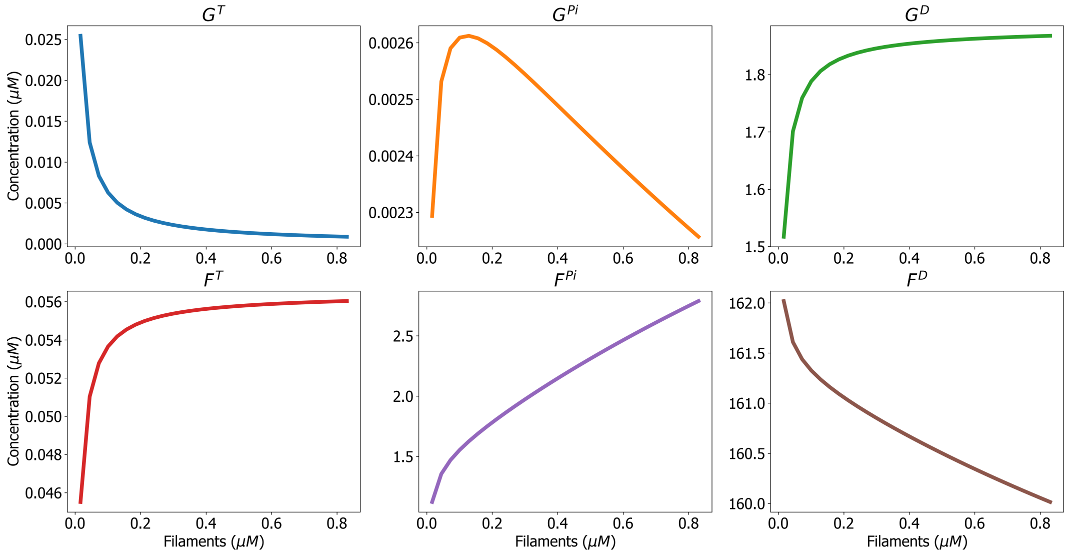

Figure 4: The concentrations at steady-state of the various actin subunit species as the concentration of filaments N_{fil} is varied. These curves have no dependence on initial conditions, except F^D which will increase linearly with the total concentration of actin subunits M; any additional actin subunits in the system will accumulate in the form F^D at steady-state.

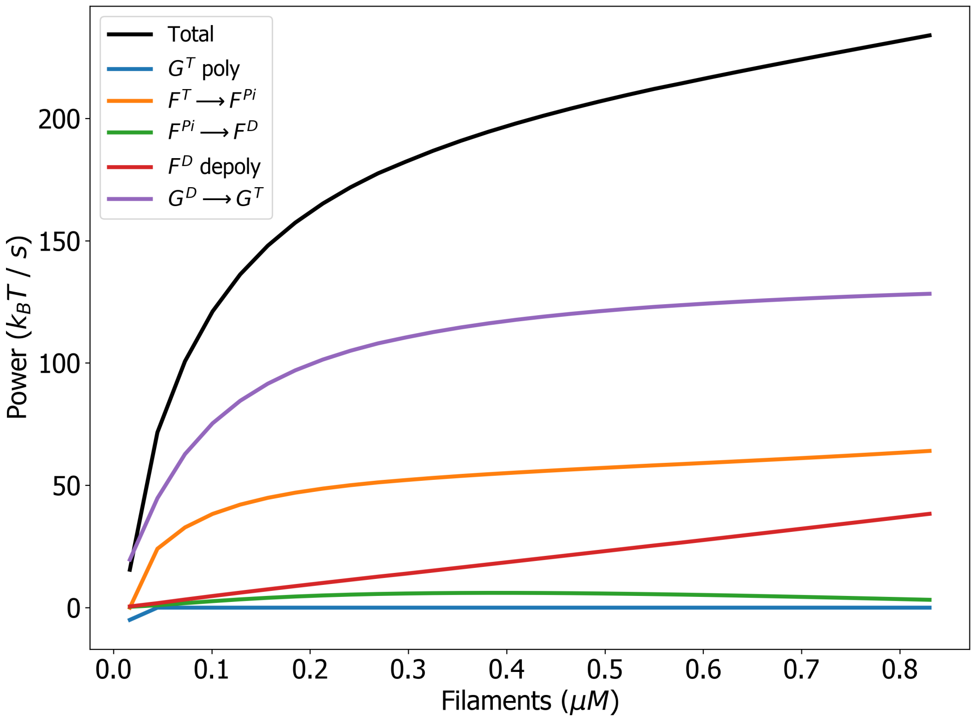

Figure 5: Contributions of each reaction in the main treadmilling pathway to the total dissipation rate at steady-state, as the concentration of filaments N_{fil} is varied. These curves have no dependence on the initial concentrations of the different subunit species.

2 Supplementary Figures

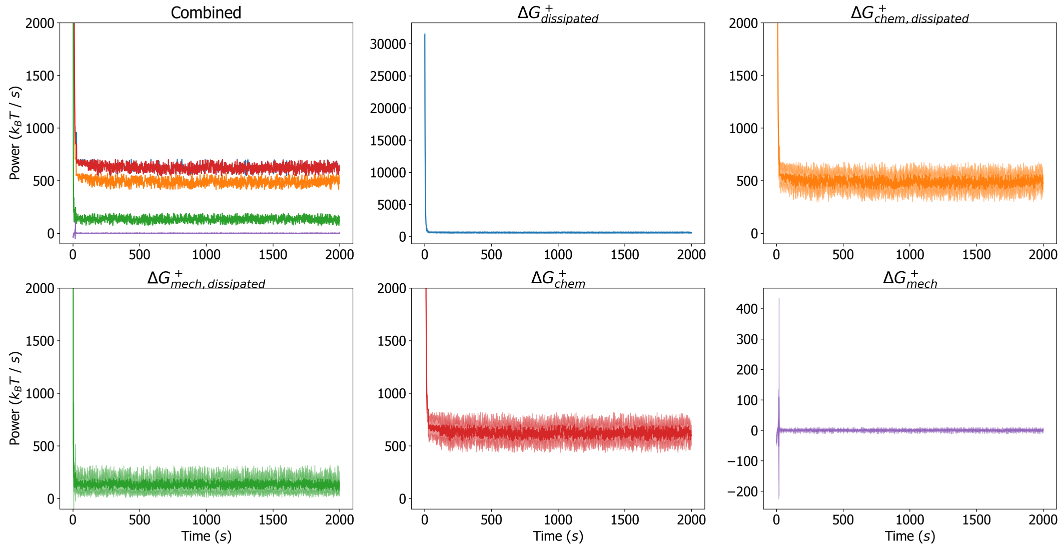

Figure 6: *Top Left*: Combined trajectories of 5 quantities tracked during a MEDYAN simulation, averaged over 10 separate runs. The color coding is indicated by the remaining panels. In these simulations, $C_{CL} = 0.1\ \mu M$ and $C_M = 0.1\ \mu M$, i.e. both concentrations are the lowest of the different conditions used in this study. In the remaining panels, the individual trajectories are visualized with their standard deviations at each time point over the 10 runs visualized as as lighter curves above and below the main curve.

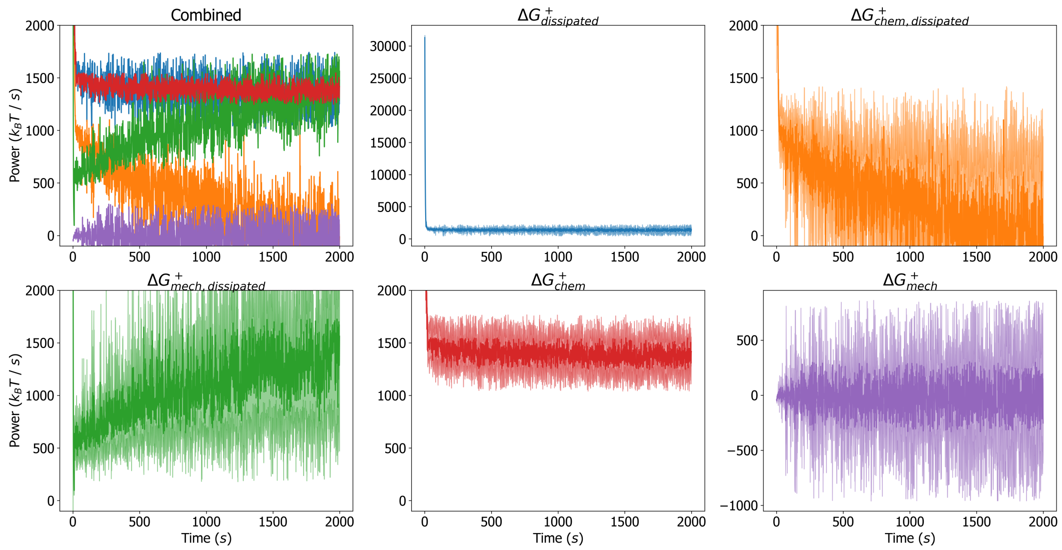

Figure 7: *Top Left*: Combined trajectories of 5 quantities tracked during a MEDYAN simulation, averaged over 10 separate runs. The color coding is indicated by the remaining panels. In these simulations, $C_{CL} = 5.0\ \mu M$ and $C_M = 0.4\ \mu M$, i.e. both concentrations are the highest of the different conditions used in this study. In the remaining panels, the individual trajectories are visualized with their standard deviations at each time point over the 10 runs visualized as as lighter curves above and below the main curve.

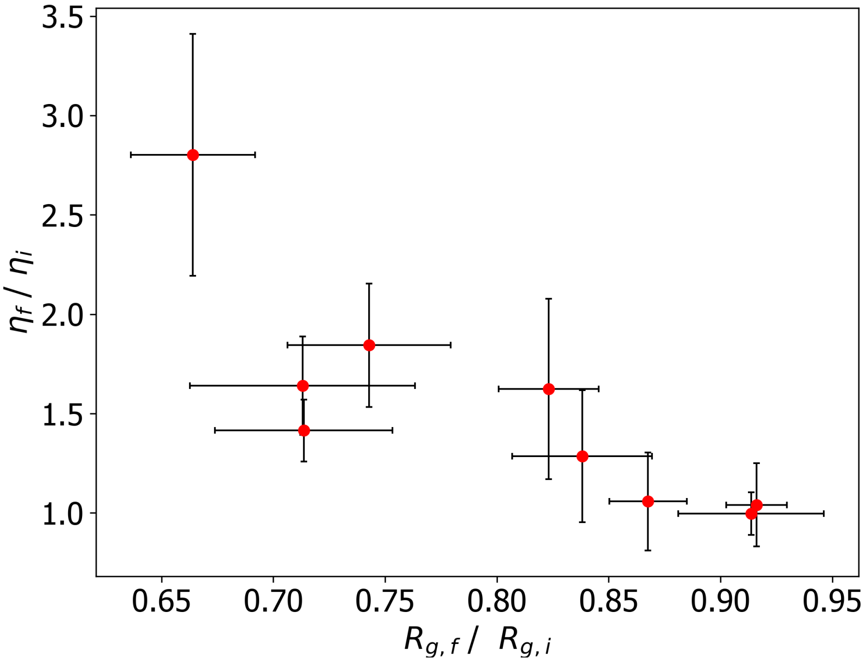

Figure 8: Networks which tend to collapse also tend to increase their ability to transduce chemical energy into mechanical stress. The change in the radius of gyration averaged over the last 100 s of a 2000 s simulation, $R_{g,f}$ divided by the radius of gyration averaged over the initial 100 s, $R_{g,i}$ measures the degree of network collapse over the trajectory. The same ratio is formed for the efficiency η, by taking the median over the same 100 s windows. These quantities are measured for each of the 9 conditions, and are averaged over the 10 repetitions of each. The error bars indicate the standard deviations over these sets of 10 measurements. The radius of gyration is defined as $R_g = \sqrt{\frac{1}{n}\sum_{i=1}^{n}(r_i - r_{GC})^2}$, where r_{GC} is the geometric center of the actin network and r_i is the position of the ith bead in the network. The efficiency is defined as $\eta = \frac{\Delta G_{\text{stress}}}{\Delta G^{+}_{\text{chem}}}$.

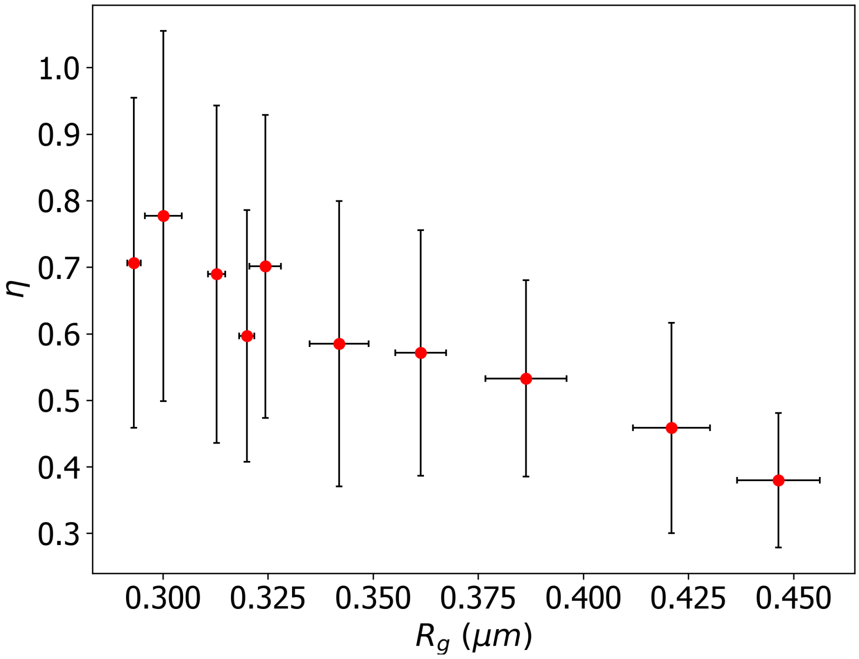

Figure 9: Efficiency is correlated with R_g in networks which exhibit collapse. A single trajectory of 2000 s with $C_{CL} = 5.0$ μM and $C_M = 0.4$ μM is divided into 10 windows. The median of η and the average of R_g is determined in each window, as well as the MAD and standard deviations, respectively, which are represented as the error bars.

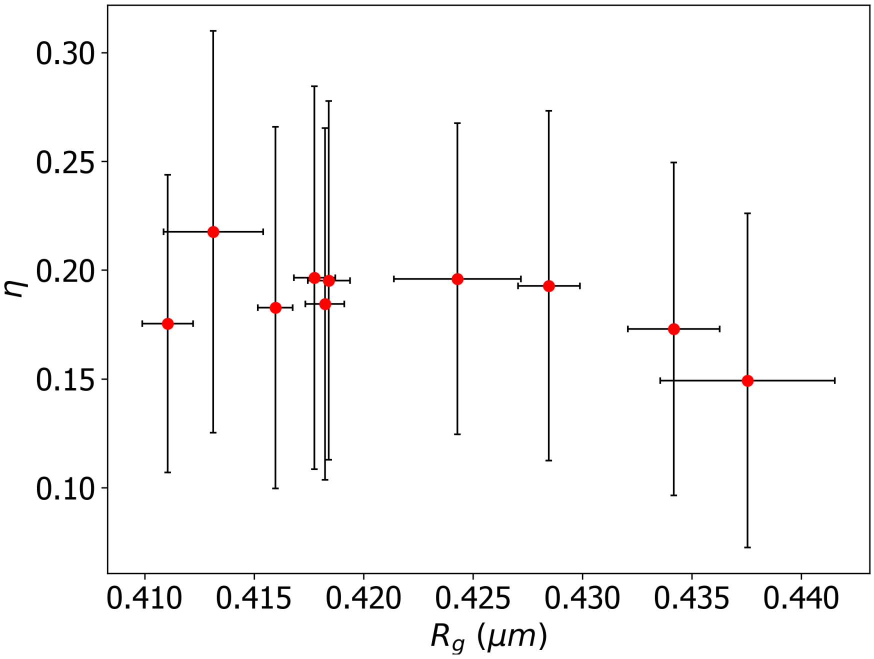

Figure 10: Efficiency is uncorrelated with R_g in networks which do not exhibit collapse. A single trajectory of 2000 s with $C_{CL} = 0.1\ \mu M$ and $C_M = 0.1\ \mu M$ is divided into 10 windows. The median of η and the average of R_g is determined in each window, as well as the MAD and standard deviations, respectively, which are represented as the error bars.

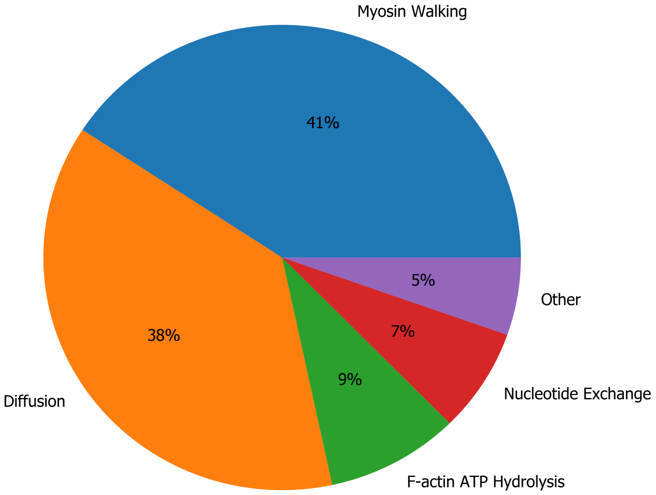

Figure 11: Integrated contributions of each reaction in MEDYAN to the total ΔG_{chem} along a simulation trajectory with $C_{CL} = 0.1\ \mu M$ and $C_M = 0.1\ \mu M$.

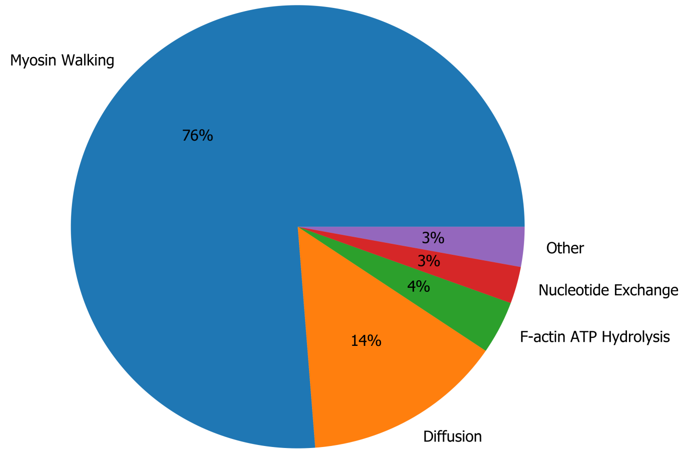

Figure 12: Integrated contributions of each reaction in MEDYAN to the total ΔG_{chem} along a simulation trajectory with $C_{CL} = 5.0\ \mu M$ and $C_M = 0.4\ \mu M$.

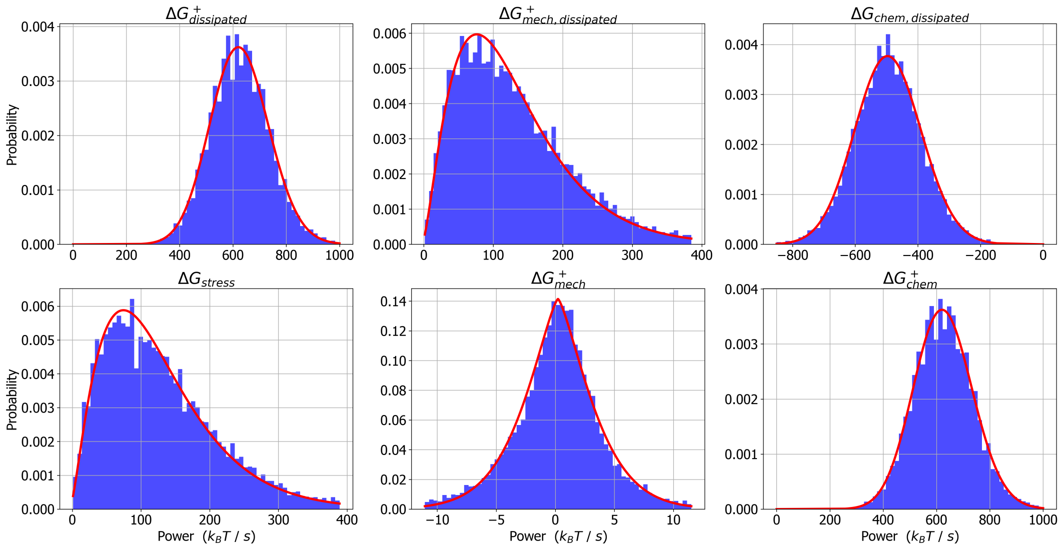

Figure 13: Histograms and fitted probability distribution functions for 6 tracked quantities. In these simulations, $C_{CL} = 0.1\ \mu M$ and $C_M = 0.1\ \mu M$, i.e. both concentrations are the lowest of the different conditions used in this study. For each histogram, the full trajectory for each of 10 runs is combined into a single data set. A log-normal distribution is used to fit each histogram with the exception of $\Delta G^+_{\mathrm{mech}}$, which is fitted with a generalized normal distribution.

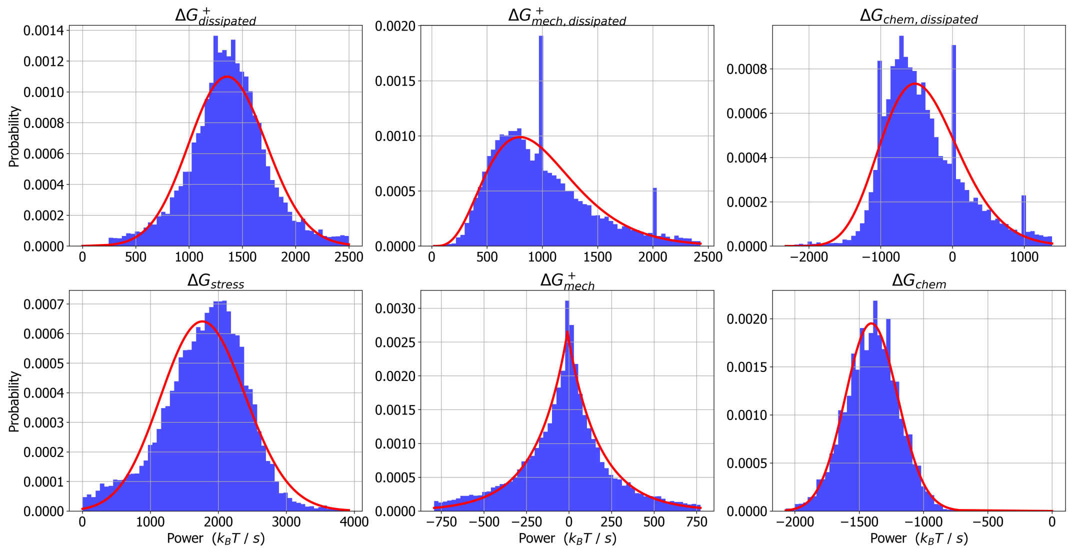

Figure 14: Histograms and fitted probability distribution functions for 6 tracked quantities. In these simulations, $C_{CL} = 5.0\ \mu M$ and $C_M = 0.4\ \mu M$, i.e. both concentrations are the highest of the different conditions used in this study. For each histogram, the full trajectory for each of 10 runs is combined into a single data set. A log-normal distribution is used to fit each histogram with the exception of ΔG^+_{mech}, which is fitted with a generalized normal distribution.

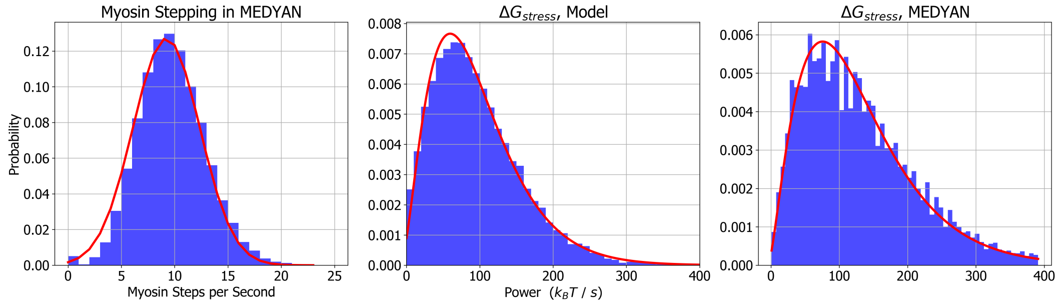

Figure 15: A simple model to qualitatively explain the shape of the distribution for the ΔG_{stress} rate at for the condition of $C_{CL} = 0.1\ \mu M$, $C_M = 0.1\ \mu M$. *Left*: Histogram of the number of myosin filament steps per second measured over a set of 10 trajectories of 2000 s each. The fitted curve shows the normal approximation to this distribution. *Middle*: The fitted normal distribution approximating the number of myosin filament steps per second ($\mu = 9.22$, $\sigma = 3.13$) is sampled from 20,000 times. Each sample of the number of myosin steps per second is squared and multiplied by a constant $\alpha \sim 1$, giving a sample of ΔG_{stress} for a one second window. This procedure models the increase in mechanical stress by a spring that increases its length beyond equilibrium by a normally distributed of fixed-length steps each second. The resulting histogram is decently fit by a gamma distribution. *Right*: For comparison, the measured histogram of ΔG_{stress}. This drastically oversimplified model of stress generation in actomyosin networks thus captures qualitative features describing the shape of this distribution.

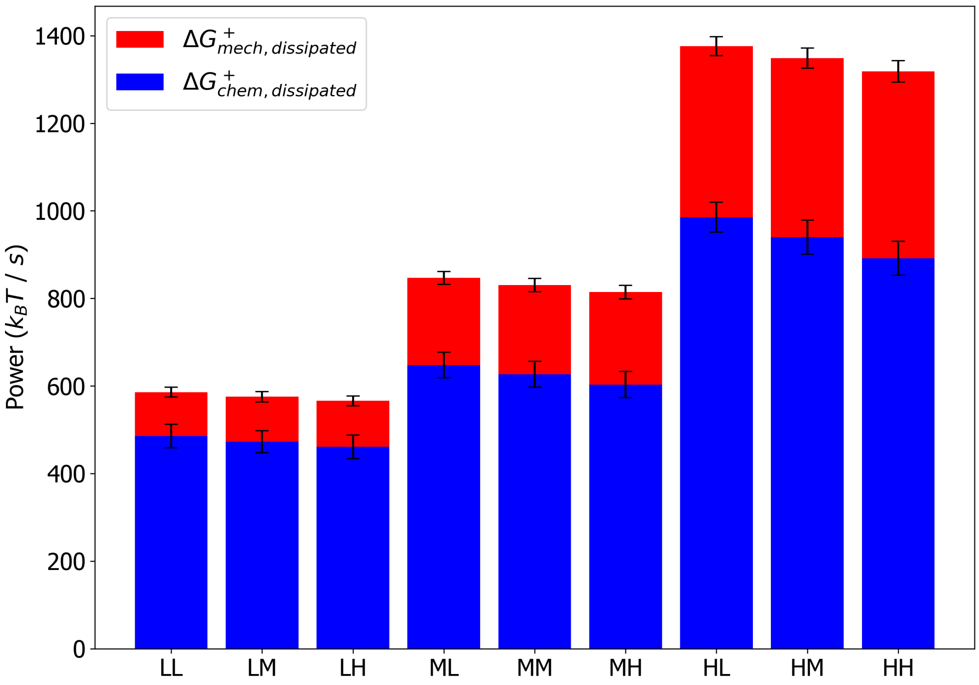

Figure 16: Bar plot representing the contributions of $\Delta G^+_{\text{chem, dissipated}}$ and $\Delta G^+_{\text{mech, dissipated}}$ to the total, $\Delta G^+_{\text{dissipated}}$, in simulations for which mechanochemical feedback is included. The letters in the abscissa labels designate "low," "medium," and "high." The first letter represents the concentration of myosin, C_M: "L" $= 0.1$ μM, "M" $= 0.2$ μM, "H" $= 0.4$ μM, and the second letter represents the concentration of cross-linkers, C_{CL}: "L" $= 0.1$ μM, "M" $= 1.0$ μM, "H" $= 5.0$ μM. The median of each quantity is taken over 10 runs of 2000 s, and error bars represent 1 MAD.

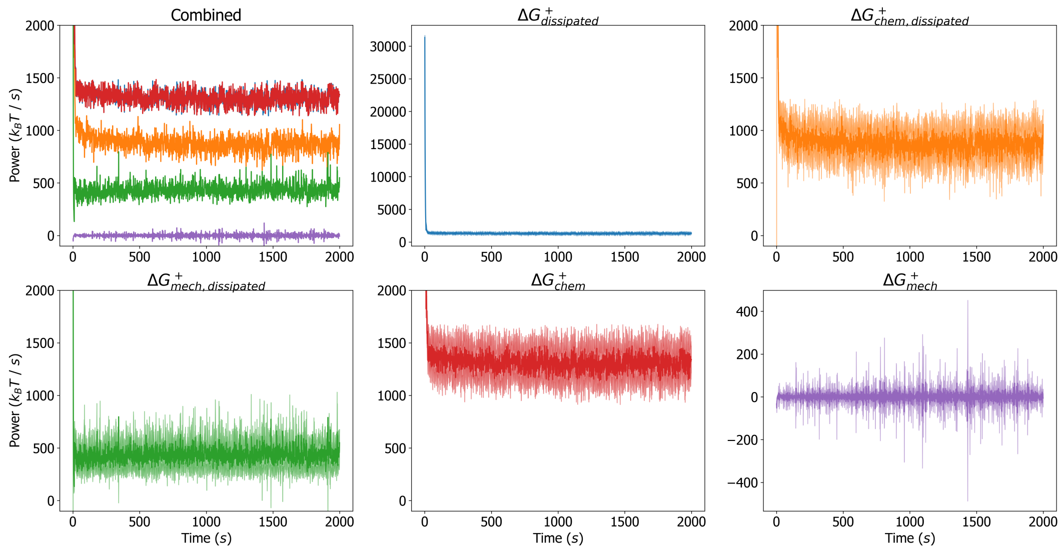

Figure 17: *Top Left*: Combined trajectories of 5 quantities tracked during a MEDYAN simulation, averaged over 10 separate runs, in simulations for which mechanochemical feedback is included. The color coding is indicated by the remaining panels. In these simulations, $C_{CL} = 5.0\ \mu M$ and $C_M = 0.4\ \mu M$, i.e. both concentrations are the highest of the different conditions used in this study. In the remaining panels, the individual trajectories are visualized with their standard deviations at each time point over the 10 runs visualized as as lighter curves above and below the main curve.

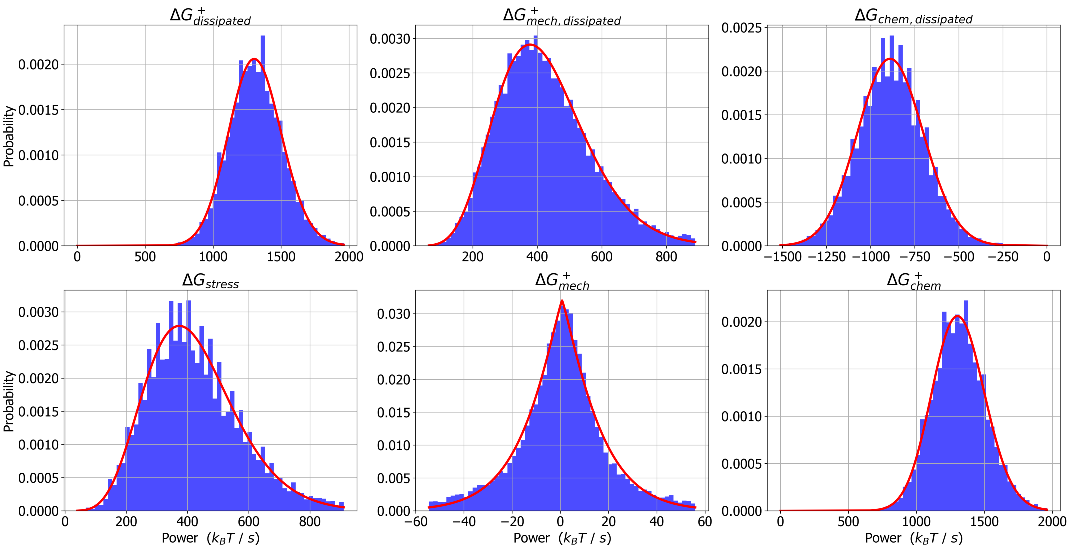

Figure 18: Histograms and fitted probability distribution functions for 6 tracked quantities, in simulations for which mechanochemical feedback is included. In these simulations, $C_{CL} = 5.0$ μM and $C_M = 0.4$ μM, i.e. both concentrations are the highest of the different conditions used in this study. For each histogram, the full trajectory for each of 10 runs is combined into a single data set. A log-normal distribution is used to fit each histogram with the exception of ΔG^+_{mech}, which is fitted with a generalized normal distribution.

References

[1] Carlos Floyd, Garegin A. Papoian, and Christopher Jarzynski. A Discrete Approximation to Gibbs Free Energy of Chemical Reactions is Needed for Accurately Calculating Entropy Production in Mesoscopic Simulations. *arXiv e-prints*, page arXiv:1901.10520, January 2019.

[2] FJ Brooks and AE Carlsson. Nonequilibrium actin polymerization treated by a truncated rate-equation method. *Physical Review E*, 79(3):031914, 2009.

[3] Andrzej Duda and Adam Kowalski. Thermodynamics and kinetics of ring-opening polymerization. *Handbook of ring-opening polymerization*, pages 1–51, 2009.

[4] Thorsten Erdmann, Philipp J Albert, and Ulrich S Schwarz. Stochastic dynamics of small ensembles of non-processive molecular motors: The parallel cluster model. *The Journal of chemical physics*, 139(17):11B604_1, 2013.

[5] Thorsten Erdmann, Kathrin Bartelheimer, and Ulrich S Schwarz. Sensitivity of small myosin ii ensembles from different isoforms to mechanical load and atp concentration. *Physical Review E*, 94(5):052403, 2016.

[6] Jonathon Howard. *Mechanics of motor proteins and the cytoskeleton.* Sinauer associates Sunderland, MA, 2001.

[7] Takeshi Sakamoto, Martin R Webb, Eva Forgacs, Howard D White, and James R Sellers. Direct observation of the mechanochemical coupling in myosin va during processive movement. *Nature*, 455(7209):128, 2008.

[8] David Keller and Carlos Bustamante. The mechanochemistry of molecular motors. *Biophysical Journal*, 78(2):541–556, 2000.

[9] Paul A Dufort and Charles J Lumsden. How profilin/barbed-end synergy controls actin polymerization: A kinetic model of the atp hydrolysis circuit. *Cytoskeleton*, 35(4):309–330, 1996.

[10] Elena G Yarmola, Dmitri A Dranishnikov, and Michael R Bubb. Effect of profilin on actin critical concentration: a theoretical analysis. *Biophysical journal*, 95(12):5544–5573, 2008.

[11] Ikuko Fujiwara, Dimitrios Vavylonis, and Thomas D Pollard. Polymerization kinetics of adp-and adp-pi-actin determined by fluorescence microscopy. *Proceedings of the National Academy of Sciences*, 104(21):8827–8832, 2007.

[12] Martin McCullagh, Marissa G Saunders, and Gregory A Voth. Unraveling the mystery of atp hydrolysis in actin filaments. *Journal of the American Chemical Society*, 136(37):13053–13058, 2014.

[13] Xuejun C Zhang and Wei Feng. Thermodynamic aspects of atp hydrolysis of actomyosin complex. *Biophysics reports*, 2(5-6):87–94, 2016.

[14] Ewa Nowak and Roger S Goody. Kinetics of adenosine 5'-triphosphate and adenosine 5'-diphosphate interaction with g-actin. *Biochemistry*, 27(23):8613–8617, 1988.

[15] HJ Kinosian, LA Selden, JE Estes, and LC Gershman. Nucleotide binding to actin. cation dependence of nucleotide dissociation and exchange rates. *Journal of Biological Chemistry*, 268(12):8683–8691, 1993.

[16] Lynn A Selden, Henry J Kinosian, James E Estes, and Lewis C Gershman. Impact of profilin on actin-bound nucleotide exchange and actin polymerization dynamics. *Biochemistry*, 38(9):2769–2778, 1999.

[17] Konstantin Popov, James Komianos, and Garegin A Papoian. Medyan: mechanochemical simulations of contraction and polarity alignment in actomyosin networks. *PLoS computational biology*, 12(4):e1004877, 2016.

[18] Ron Milo and Rob Phillips. *Cell biology by the numbers.* Garland Science, 2015.

[19] Carlos Floyd, Christopher Jarzynski, and Garegin Papoian. Low-dimensional manifold of actin polymerization dynamics. *New Journal of Physics*, 19(12):125012, 2017.

[20] G Nicolis and I Prigogine. I (1977) self-organization in nonequilibrium systems. *From Dissipative structures to Order through Fluctuations, Mir, Moscow, Russia.*

[21] Dilip Kondepudi and Ilya Prigogine. *Modern thermodynamics: from heat engines to dissipative structures.* John Wiley & Sons, 2014.

[22] Ilya Prigogine. Introduction to thermodynamics of irreversible processes. *New York: Interscience, 1967, 3rd ed.*, 1967.

[23] P Glansdorff and I Prigogine. Sur les propriétés différentielles de la production d'entropie. *Physica*, 20(7-12):773–780, 1954.

[24] Charles S Peskin, Garrett M Odell, and George F Oster. Cellular motions and thermal fluctuations: the brownian ratchet. *Biophysical journal*, 65(1):316–324, 1993.
